# Role of the E3 ubiquitin-ligase Hakai in intestinal inflammation and cancer bowel disease

**DOI:** 10.1038/s41598-022-22295-w

**Published:** 2022-10-20

**Authors:** Daniel Roca-Lema, Macarena Quiroga, Vineeta Khare, Andrea Díaz-Díaz, Aida Barreiro-Alonso, Andrea Rodríguez-Alonso, Ángel Concha, Gabriela Romay, M. Esperanza Cerdán, Christoph Gasche, Angélica Figueroa

**Affiliations:** 1 Epithelial Plasticity and Metastasis Group, Instituto de Investigación Biomédica de A Coruña (INIBIC), Complexo Hospitalario Universitario de A Coruña (CHUAC), Sergas, Universidade da Coruña (UDC), As Xubias, 15006 A Coruña, Spain; 2 Division of Gastroenterology and Hepatology, Department of Internal Medicine III, Medical University of Vienna, Vienna, Austria; 3 Functional Proteomics Group, Chester Beatty Laboratories, Institute of Cancer Research, London, UK; 4 EXPRELA, Centro de Investigacións Científicas Avanzadas (CICA), Departamento de BioloxíaFacultade de Ciencias, Universidade da Coruña, Campus da Zapateira, A Coruña, Spain; 5 Pathology Department and A Coruña Biobank From INIBIC, CHUAC, Sergas, UDC, A Coruña, Spain

**Keywords:** Cancer, Cell biology, Molecular biology, Diseases

## Abstract

The E3 ubiquitin-ligases are important for cellular protein homeostasis and their deregulation is implicated in cancer. The E3 ubiquitin-ligase Hakai is involved in tumour progression and metastasis, through the regulation of the tumour suppressor E-cadherin. Hakai is overexpressed in colon cancer, however, the implication in colitis-associated cancer is unknown. Here, we investigated the potential role of Hakai in intestinal inflammation and cancer bowel disease. Several mouse models of colitis and associated cancer were used to analyse Hakai expression by immunohistochemistry. We also analysed Hakai expression in patients with inflamed colon biopsies from ulcerative colitis and Crohn's disease. By Hakai interactome analysis, it was identified Fatty Acid Synthase (FASN) as a novel Hakai-interacting protein. Moreover, we show that Hakai induces FASN ubiquitination and degradation via lysosome, thus regulating FASN-mediated lipid accumulation. An inverse expression of FASN and Hakai was detected in inflammatory AOM/DSS mouse model. In conclusion, Hakai regulates FASN ubiquitination and degradation, resulting in the regulation of FASN-mediated lipid accumulation, which is associated to the development of inflammatory bowel disease. The interaction between Hakai and FASN may be an important mechanism for the homeostasis of intestinal barrier function and in the pathogenesis of this disease.

## Introduction

Inflammation is an important risk factor for the development of human cancers. Colitis-associated colorectal cancer (CAC) is a specific type of colorectal cancer developed from colitis in inflammatory bowel disease (IBD) patients. IBD, including ulcerative colitis (UC) and Crohn’s disease (CD), is characterized by chronic inflammation in the gastrointestinal tract and can induce pre-neoplastic lesions, being an important risk factor for the onset of colorectal cancer^1,2^. Ubiquitination is one of the most important translational modifications in eukaryotes that induces substrate degradation, which in consequence controls the “quantity” and “quality” of specific proteins, ensuring cell homeostasis^3,4^. Ubiquitination process consists in the ubiquitin moiety linkage to a specific substrate that signals for degradation^5^. Three-enzymes are involved in the ubiquitination cascade: the E1 ubiquitin-activating enzyme that activates the ubiquitin molecule, the E2 ubiquitin-conjugating enzyme that carries the activated ubiquitin molecule as a thioester to the E3 ubiquitin-ligase enzyme that transfers the activated ubiquitin from the E2 to the lysine residue of substrate protein^5,6^. Among all RING-type E3 ligases, there are a number that require substrate phosphorylation at a tyrosine residue (pTyr) for their recognition, including Cbl family^7^. Excessive ubiquitin-mediated proteolysis has been observed in different types of cancer as well as in colitis, and its inhibition was shown to improve the colitis in the mouse model^8–10^.

Hakai protein is an E3 ubiquitin-ligase, identified as the first posttranslational regulator of the E-cadherin stability. Hakai induces E-cadherin ubiquitination and degradation in a Src-dependent manner; which in turn causes the alteration of cell–cell adhesions^11,12^. E-cadherin is the best-characterized member of adherens junctions, a type of cell–cell contacts that participate in embryogenesis and tissue homeostasis^13,14^. The loss of E-cadherin at cell–cell contacts induces the epithelial-to-mesenchymal transition (EMT), a process by which cells lose cell polarity and acquire a migratory phenotype, a fundamental program during colon tumor progression and metastasis^15–17^. In human biopsies from IBD patients, E-cadherin expression is downregulated. Indeed, E-cadherin is the strongest candidate for UC susceptibility and plays a key role in epithelial restitution and repair following mucosal damage. In fact, expression of E-cadherin is significantly reduced in areas of active UC. Of note, E-cadherin has recently been associated with susceptibility to colorectal cancer, which is an established complication of longstanding UC. The new associations suggest that changes in the integrity of the intestinal epithelial barrier may contribute to the pathogenesis of UC^18–22^. Moreover, In an in vivo mouse model of experimentally induced colitis, it is shown that E-cadherin deficiency was associated with exacerbated acute and chronic inflammation highlighting its role in the pathogenesis of UC^23^. We have previously demonstrated that Hakai is an important regulator of cell proliferation, epithelial-to-mesenchymal transition, cell invasion, and that it induces tumour progression and metastasis in vivo^24–26^. We have also shown that Hakai expression is increased in colon and gastric cancer compared to adjacent human colon healthy tissues, with important clinical implications for designing new therapeutic strategies^26–28^.

Several E3 ubiquitin-ligases were reported to be implicated in the pathogenesis and development of human IBD^8^. However, whether the E3 ubiquitin-ligase Hakai participates in IBD is still unknown. The main purpose is to explore the implication of Hakai in IBD by analyzing Hakai expression in murine models for CAC. In the present study, we have shown that Hakai is downregulated under pro-inflammatory environment in the mice intestinal tissue regardless of origin of that inflammation. Moreover, by an interactome analysis we have identified Fatty Acid Synthase (FASN) as a novel Hakai-interacting protein. Hakai induces FASN ubiquitination and degradation via lysosome, regulating FASN-mediated lipid accumulation, which is associated to the development of IBD. Importantly, an inverse association of FASN expression with Hakai expression was detected in inflammatory AOM/DSS compared to tumour tissue of CAC and healthy tissues.

## Results

### Hakai expression in different mouse models of colitis and CAC

**Figure 1:**
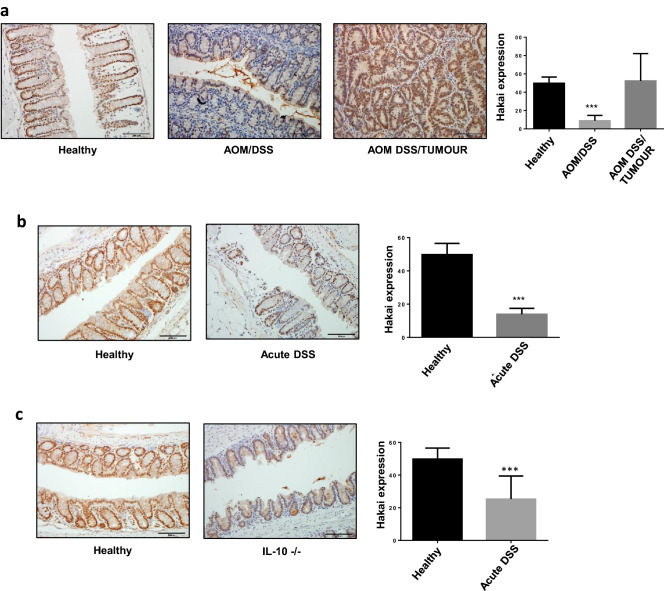
Expression of Hakai in mouse models of colitis and CAC. (**a**) Immunohistochemistry staining of Hakai in healthy non-treated mouse colonic mucosa and in gut sections of AOM/DSS-treated mouse (inflamed epithelium and tumours, healthy mice, n = 7; AOM/DSS-treated mice, n = 14)**.** (**b**) Immunohistochemistry staining of Hakai in healthy non-treated mouse colonic mucosa and in gut sections for DSS-treated mouse (inflamed epithelium, healthy mice, n = 7; DSS-treated mice, n = 3). (**c**) Immunohistochemistry staining of Hakai in healthy non-treated mouse colonic mucosa and in gut sections for IL-10 deficient mice (inflamed epithelium, healthy mice, n = 7; IL-10 deficient mice, n = 6). Images obtained with ×20 objective. Five pictures of each sample were taken and quantified. Data are represented as scatter plot. Values are means ± SEM of staining intensity signal scoring per area. Quantification and calibration of the images were performed with Immunohistochemistry Image Analysis Toolbox software for Image J. Kruskal–Wallis with Tukey correction test analyses show statistical differences in AOM/DSS-inflamed tissue respect to paired healthy samples (*p < 0.05; **p < 0,01; ***p < 0,001). Scale bar 125 μm.

Hakai overexpression in cytoplasm and nucleus was reported in human colon cancer compared to adjacent normal healthy colon tissues^24,26,27^. In order to compare Hakai expression in the context of intestinal inflammation, three different mouse models that mimic different origins and stages of the IBD disease were used. Firstly, an AOM-DSS model of CAC was used. This model replicates the tumour development process of the human CAC by using the combination of a pro-inflammatory agent (DSS) and a carcinogenic compound (AOM), as previously described^29^. As shown in Fig. 1a, the immunohistochemistry analysis of the whole intestine samples using Hakai antibody revealed a significant decreased expression of Hakai in inflammatory conditions (AOM/DSS) of the gut epithelium compared to tumour tissue from the CAC (AOM DSS/Tumour) or healthy tissues. The same pattern of expression was observed when analysing an animal model of acute colitis (acute DSS), which resemble the acute phase of the disease or flare-ups by using higher concentrations of pro-inflammatory agent DSS at short times (Fig. 1b). Finally, a third stablished mouse model was used based in a genetically modified mice deficient for the IL-10 gene (IL-10 KO). These mice spontaneously develop a chronic inflammatory bowel disease (IBD) due to the important control of IL-10 on the gut microbiota^30^. The absence of IL-10 modulates cellular immune response and reduces mice tolerance towards bacterial antigens of enteric bacteria which causes the inflammatory process. In this model, Hakai expression was also statistically reduced in inflamed conditions compared to gut epithelium of healthy mice (Fig. 1c). All these results point out to a regulation of Hakai in different inflammatory conditions, with a great reduction of Hakai expression in the inflamed epithelium. Remarkably, high expression of Hakai in the epithelium of healthy untreated mouse was detected while this observation was not shown in human tumour-adjacent healthy tissue^26^.

**Figure 2:**
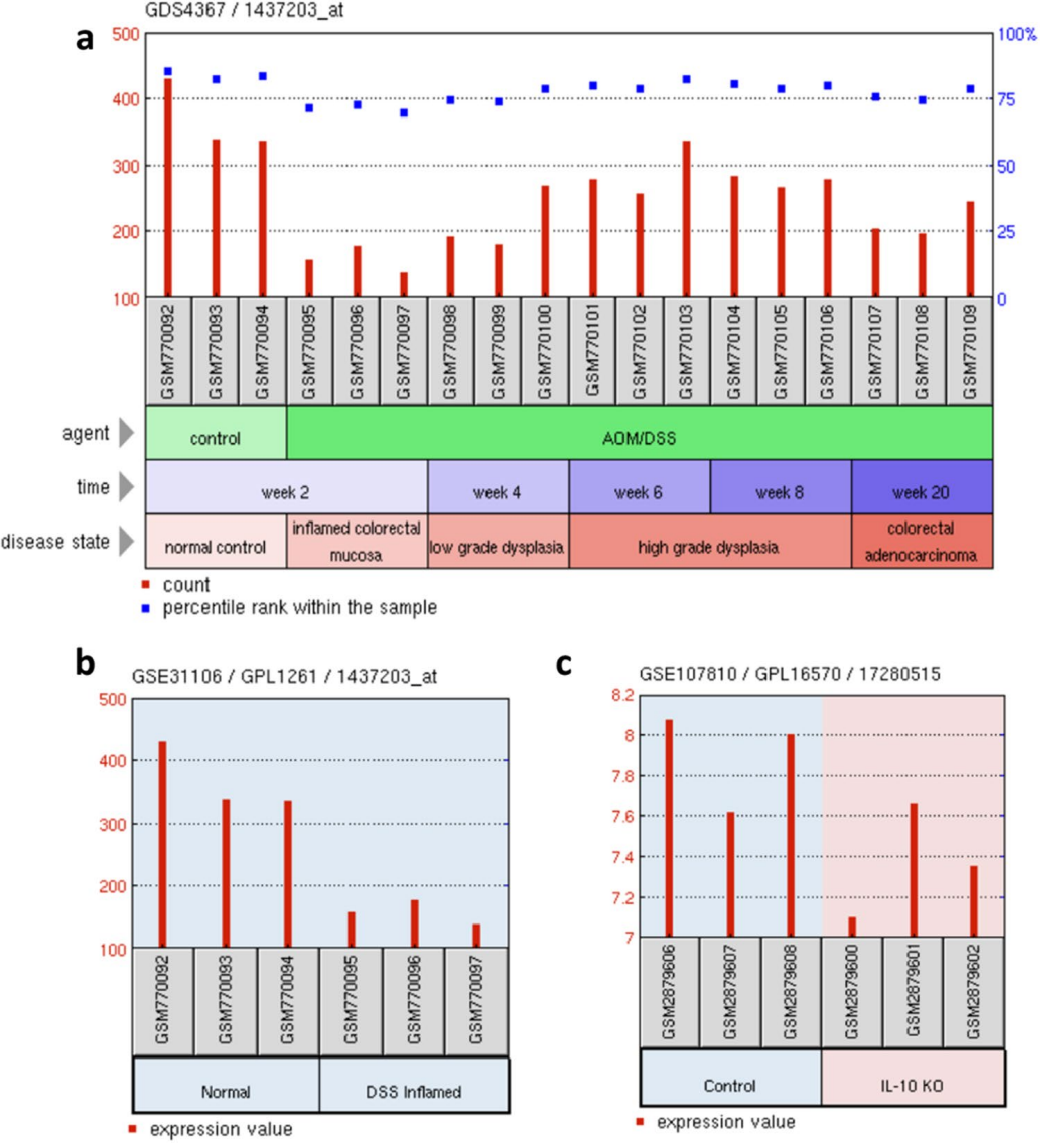
Gene expression of Hakai in AOM/DSS, acute DSS and IL-10 KO mouse models using microarray data from similar experiments in NCBI’s Gene Expression Omnibus (GEO). (**a**) Gene expression of Hakai during temporal analysis of mouse colon tissue from AOM/DSS-induced model of UC-associated colorectal cancer. (**b**) Hakai gene expression differences between normal control and DSS inflamed mucosa in mouse. (**c**) Hakai gene expression in colon tissue from IL-10 WT and IL-10 KO mice. Data from GEO database accession numbers: GDS4367, GSE31106 and GSE107810.

Then we further analysed the Hakai mRNA expression by using microarray data from similar experiments in NCBI’s Gene Expression Omnibus (GEO). Three different mouse models were analysed: the colitis-associated colorectal cancer (AOM/DSS), the acute colitis (acute-DSS), and the IL-10 KO mouse models. As shown, the levels of Hakai mRNA were reduced in the inflamed tissue in AOM/DSS model (Fig. 2a), the DSS mouse model (Fig. 2b) and the IL-10 KO mouse model (Fig. 2c). Moreover, Hakai mRNA expression was recovered during tumour progression (low grade dysplasia, high grade dysplasia and colorectal adenocarcinoma) in the AOM/DSS model (Fig. 2a). Taking together these results suggest that, mRNA and protein expression of Hakai are clearly regulated under a pro-inflammatory environment in the intestinal tissue regardless of origin of that inflammation.

### FASN as a novel Hakai-regulated protein identified by interactome analysis

**Figure 3:**
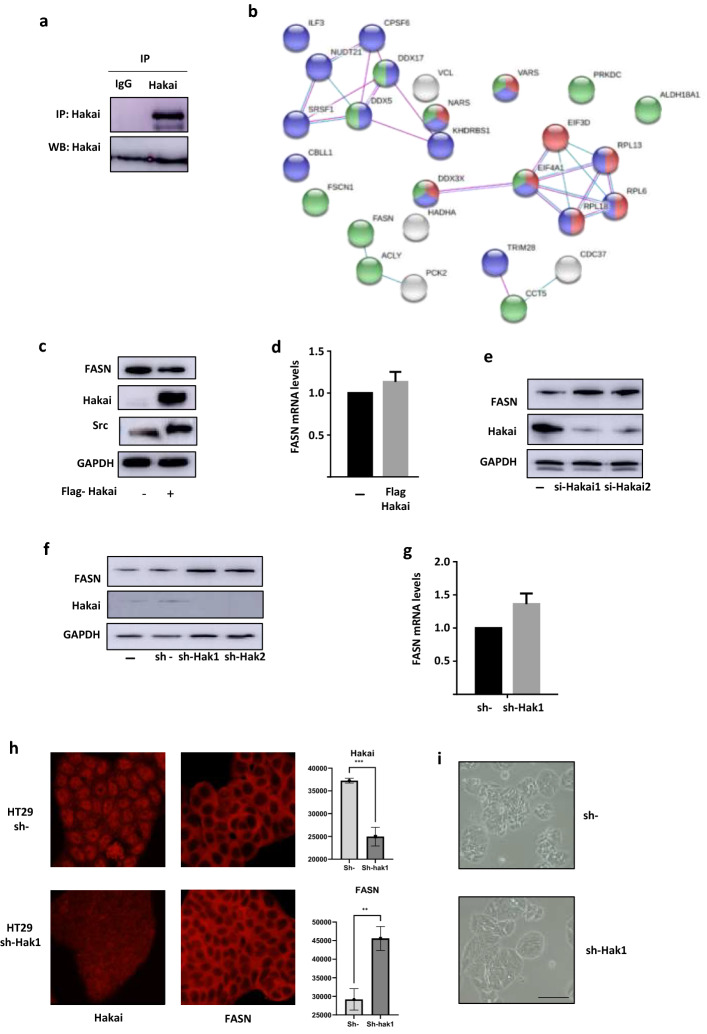
FASN is a novel identified protein in the Hakai interactome analysis. (**a**) Immunoprecipitation of endogenous Hakai in HCT116 cells. (**b**) Network using STRING database of all the identified proteins in Hakai immunoprecipitation in HCT-116. Proteins in green are related to drug binding, red colour, implication in translation and blue colour in RNA processing. (**c**) Effect of Hakai overexpression reduces FASN protein levels. Transfection with pcDNA-Flag-Hakai (2 µg) and pSG-v-Src (0.5 µg) was performed in HCT116 cells following by western blot with the indicated antibodies. (**d**) Effect of Hakai overexpression, as described in C, in FASN mRNA levels analysed by RT qPCR using RPL13A and GAPDH as control (**e**,**f**) Effect of Hakai silencing on FASN using transient transfection in HCT116 cells (**e**) or an inducible viral-transduced system with 1 µg/ml doxycycline for Hakai silencing in HT29 (**f**). Cell lysates were subjected to Western Blotting as described. (**g**) mRNA levels of FASN determined in the inducible viral-transduced system for Hakai silencing in HT29, analysed by RT qPCR using RPL13A and GAPDH as a housekeeping controls. (**h**) Effect of Hakai silencing in FASN subcellular localization in the inducible viral-transduced system in HT29 cells. Immunofluorescence images with anti-Hakai and anti-FASN antibodies (left panel) and quantification (right panel) is included. Images taken with 40X magnification. (**i**) Effect of Hakai on phenotype by using an inducible silencing viral system on HT29 cell. Optical microscopy images taken after 72 h induction. Scale bar 125 μm. The blots images were cropped prior hybridization and original blots are included in Supplementary Information.

In order to understand the potential molecular mechanism by which Hakai could be involved in inflammation, HCT116 colon cell line was used to perform a large-scale immunoprecipitation of Hakai followed by a mass spectrometric analysis in order to determine novel Hakai-interacting proteins that could be related to inflammatory process. For Hakai interactome, immunoprecipitation of endogenous Hakai protein levels was firstly confirmed (Fig. 3a). A total of 26 proteins with a peptide match ≥ 2 were identified by proteomic analysis in Hakai immunoprecipitated samples compared to non-specific immunoprecipitation using IgG control (Table 1). It should be noted that, Hakai is among the identified proteins and also HSP90 protein, another previously Hakai-interacting protein reported^31^, ensuring the specificity of the developed technique. A map of Hakai molecular interactions was performed by using the biological database STRING that provide information about both physical and functional properties of proteins (Fig. 3b)^32^. Interestingly, the Fatty Acid Synthase (FASN) protein was found as a novel Hakai-interacting protein. FASN is a multifunctional enzyme that catalyses the novo synthesis of long chain saturated fatty acids, from acetyl-CoA and malonyl-CoA, in the presence of NADPH^33^. Long-chain fatty acids considerably contribute to the pathology of intestinal epithelial cells, however, under normal conditions, the synthesis of fatty acids takes place in order to store energy. Abnormalities of lipid metabolism through overexpression of FASN, are associated with the development of inflammatory bowel disease (IBD)^34,35^. On the other hand, FASN expression has been detected in some benign and preneoplastic lesions of several organs such as colon, prostate, breast, lung, stomach, and skin nevi^36,37^.

**Table 1:** List of identified proteins in Hakai interactome analysis.

N	Accession number	Gene code	Protein name	Biological role
1	Q9Z1X4	ILF3	Interleukin enhancer-binding factor 3	Transcription factor (NF45, ILF2) required for T-cell expression of interleukin 2
2	Q16630	CPSF6	Cleavage and polyadenylation specificity factor subunit 6	Subunit of a cleavage factor required for 3' RNA cleavage and polyadenylation processing
3	O43809	NUDT21	Cleavage and polyadenylation specificity factor subunit 5	Subunit of a cleavage factor required for 3' RNA cleavage and polyadenylation processing
4	Q92841	DDX17	ATP-dependent RNA helicase DDX17	RNA helicases. They are implicated in a number of cellular processes involving alteration of RNA secondary structure
5	P17844	DDX5	ATP-dependent RNA helicase DDX5	RNA helicases. This protein is involved in pathways that include the alteration of RNA structures
6	Q07955	SRSF1	Serine/arginine-rich splicing factor 1	Arginine/serine-rich splicing factor protein family
7	Q16658	FSCN1	Fascin	Member of the fascin family of actin-binding proteins
8	P18206	VCL	Vinculin	Cytoskeletal protein associated with cell–cell and cell–matrix junctions
9	Q2KJG3	NARS	Asparagine tRNA ligase	Asparaginyl-tRNA synthetase
10	Q07666	KHDRBS1	KH domain-containing, RNA-binding, signal transductionassociated protein 1	Member of the K homology domain-containing, RNA-binding, signal transduction-associated protein family
11	P26640	VARS	Valine tRNA ligase	Valyl-tRNA synthetase
12	O00571	DDX3X	ATP-dependent RNA helicase DDX3X	ATP-dependent RNA helicase
13	P40939	HADHA	Trifunctional enzyme subunit alpha	Catalyzes the last three steps of mitochondrial beta-oxidation of long chain fatty acids
14	P49327	FASN	Fatty acid synthase	Catalyze the synthesis of palmitate from acetyl-CoA and malonyl-CoA, in the presence of NADPH, into long-chain saturated fatty acids
15	P53396	ACLY	ATP-citrate synthase	Primary enzyme responsible for the synthesis of cytosolic acetyl-CoA in many tissues
16	Q16822	PCK2	Phosphoenolpyruvate carboxykinase	Mitochondrial enzyme that catalyzes the conversion of oxaloacetate to phosphoenolpyruvate in the presence of guanosine triphosphate
17	Q13263	TRIM28	Transcription intermediary factor 1-beta	Gene mediates transcriptional control by interaction with the Kruppel-associated box repression domain found in many transcription factors
18	P48643	CCT5	T-complex protein 1 subunit epsilon	Molecular chaperone that is a member of the chaperonin containing TCP1 complex
19	Q16543	CDC37	Hsp90 co-chaperone	Molecular chaperone with specific function in cell signal transduction. It has been shown to form complex with Hsp90
20	P78527	PRKDC	DNA-dependent protein kinase catalytic subunit	Catalytic subunit of the DNA-dependent protein kinase (DNA-PK). It functions with the Ku70/Ku80 heterodimer protein in DNA double strand break repair and recombination
21	P54886	ALDH18A1	Delta-1-pyrroline-5-carboxylate synthase	Catalyzes the reduction of glutamate to delta1-pyrroline-5-carboxylate, a critical step in the de novo biosynthesis of proline, ornithine and arginine
22	O15371	EIF3D	Eukaryotic translation initiation factor 3 subunit D	The complex binds to the 40S ribosome and helps maintain the 40S and 60S ribosomal subunits in a dissociated state
23	P26373	RPL13	60S ribosomal protein L13	This gene encodes a ribosomal protein that is a component of the 60S subunit
24	Q02878	RPL6	60S ribosomal protein L6	This gene encodes a protein component of the 60S ribosomal subunit
25	Q07020	RPL18	60S ribosomal protein L18	Ribosomes, the organelles that catalyze protein synthesis, consist of a small 40S subunit and a large 60S subunit
26	P60842	EIF4A1	Eukaryotic initiation factor 4A-I	Eukaryotic translation initiation factor 4A1

In order to determine the possible link between Hakai and FASN, HCT116 cells were transfected with Hakai, in presence of Src, and the effect on FASN expression levels was assessed. Hakai overexpression strongly reduced FASN protein expression in HCT-116 cells, while no effect was detected at mRNA levels (Fig. 3c,d), further suggesting that Hakai may regulate FASN at a post-transcriptional level. Then, we tested the effect of Hakai-silencing on FASN expression. Transient transfections of two different oligos of siRNA Hakai in HCT116 cells and viral transduction of shRNA-Hakai silencing in an inducible system in HT29 were performed. Hakai silencing strongly increased FASN protein expression in both cell lines (Fig. 3e,f), while mRNA expression of FASN was not affected by Hakai silencing (Fig. 3g), further supporting the effect of Hakai on FASN at post-transcriptional level. Then it was analysed the effect of Hakai-silencing in the localization of FASN by immunofluorescence. The strong reduction of Hakai in the cells after inducing the knockdown with doxycycline confirmed the increased of FASN, without affecting FASN cellular localization (Fig. 3h). At the phenotypic level, silencing of Hakai induced a more epithelial phenotype, with cells closer attached between each other accompanied to a more three-dimensional cell sheet, compared to control conditions (Fig. 3i).

### Hakai is a post-translational regulator of FASN via ubiquitin-lysosome pathway

**Figure 4:**
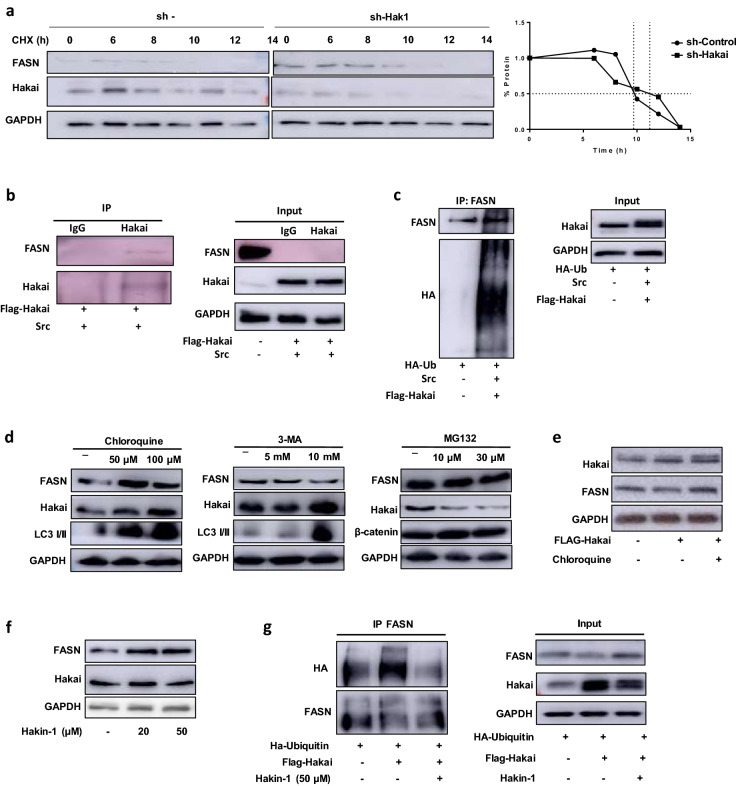
Hakai regulates ubiquitination and degradation of FASN via lysosome. (**a**) Analysis of FASN degradation by cycloheximide chase. FASN half-life in HT29 cell line was determined by treatment with 10 µg/ml Cycloheximide for the indicated time course and compared with FASN half-life in Hakai-silenced HT29 cells compared to control HT29 cells. Hakai-silencing was performed by viral transduction induced with 1 µg/ml of Doxycycline for 72 h. Quantification of endogenous FASN protein half-life are shown (right panel). (**b**) Coimmunoprecipitation of Hakai and FASN. Hakai was immunoprecipitated in HCT-116 cells transfected with pcDNA-Flag-Hakai and v-Src. Hakai immunoprecipitation was performed using anti-Hakai antibody. Coimmunoprecipitation was evaluated by western blot as described in materials and methods using the indicated antibodies. GAPDH signal was used as protein loading control. (**c**) Hakai-dependent ubiquitination of FASN Flag-Hakai, v-Src and HA-ubiquitin were transiently transfected into HCT116 cells. Immunoprecipitation was performed with the anti-FASN antibody before Western blotting, using the indicated antibodies. (**d**) FASN and Hakai levels in HCT-116 cells treated with the lysosome degradation inhibitor Chloroquine, the autophagy inhibitor 3-Methyladenine and the proteasome inhibitor MG132 at the indicated concentration times described in materials and methods. LC3 I/II levels were analyzed as a positive control of chloroquine and 3-MA treatment and β-catenin for MG132 treatment. (**e**) HCT116 cells were transiently transfected with pcDNA-Flag-Hakai and the day after transfection cells were treated with chloroquine at 50 µM for 24 h. (**f**) Effect of Hakin-1 inhibitor on FASN expression. Hakin-1 was used at increasing concentrations for 48 h in HCT-116 cells. (**g**) Effect of Hakin-1 on Hakai-dependent ubiquitination of FASN. Flag-Hakai and HA-ubiquitin were transiently transfected into HCT116 cells. Immunoprecipitation was performed with the anti-FASN antibody before Western blotting using the indicated antibodies. GAPDH was used as protein loading control. The blots images were cropped prior hybridization and original blots are included in Supplementary Information.

Given that our results suggested that Hakai may control FASN at post-transcriptional level, inhibition of protein synthesis with cycloheximide (CHX) was performed in order to determine the half-life of FASN protein in presence or absence of Hakai (Fig. 4a, left and middle panel). FASN is a rather stable protein in HT29 cell, with an approximate half-life of 9.5 h (Fig. 4a, right panel). FASN half-life was increased to 11.5 h in Hakai-silencing conditions, suggesting Hakai effect on FASN protein stability. We then analysed whether Hakai may act as an E3 ubiquitin-ligase for FASN inducing its ubiquitination and degradation. After Hakai overexpression, an interaction between Hakai and FASN was detected (Fig. 4b), further confirming the results obtained by the interactome analysis (Fig. 3b). Moreover, Hakai induced FASN ubiquitination when transfecting cells in presence of pcDNA-Flag-Hakai, pBSSR-HA-ubiquitin and pSG-v-Src (Fig. 4c). This effect was also detected when Src was not overexpressed (Fig. S1). Taken together these results indicate that Hakai binds to FASN and induces its ubiquitination and degradation in vitro. There are three described pathways by which ubiquitinated substrates can be degraded: proteasome degradation, lysosome degradation and autophagy system. Treatment with the lysosome inhibitor chloroquine did increase FASN protein levels in HCT116 cells, while the autophagy inhibitor 3-methyladenine (3-MA) did not (Fig. 4d). Given that both inhibitors exert their inhibitory function in the autophagy pathway, 3-MA at early stages of autophagosome formation and chloroquine at late-autophagy stage targeting the lysosome^38^, their eeffectiveness were confirmed by using LC3 I/II levels as positive control. Furthermore, the 26S proteasome inhibitor MG132 did not increase FASN protein (Fig. 4d), suggesting that FASN degradation does occur via lysosome degradation. Besides, FASN levels were recovered after Hakai overexpression when lysosome inhibitor chloroquine was present (Fig. 4e), indicating that Hakai induced FASN degradation is, at least in part, via lysosome. Finally, we used a specific reported Hakai inhibitor, Hakin-1, to inhibit Hakai-mediated ubiquitination without affecting Hakai protein levels^28^. As shown, Hakin-1 was able to increase FASN protein levels without affecting Hakai protein levels, further supporting Hakai-mediated action on FASN (Fig. 4f). We also observed a reduction of Hakai-dependent ubiquitination of FASN when cells were treated with Hakin-1 (Fig. 4g). Collectively, our data support that Hakai control FASN ubiquitination and degradation via lysosome.

### Hakai controls lipid accumulation via FASN

**Figure 5:**
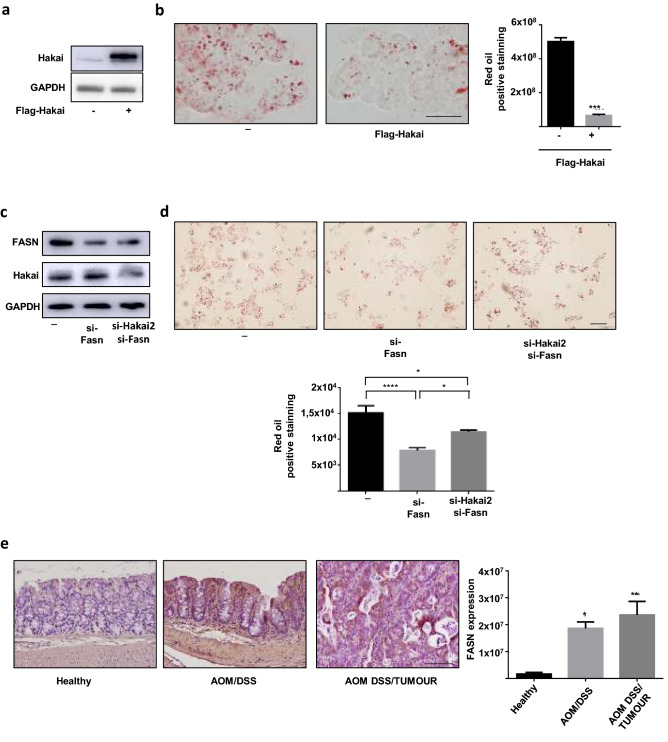
Hakai controls lipid accumulation via FASN. (**a**) Hakai expression in HCT116 cells transfected with Flag-Hakai was analyzed by Western blot analysis and (**b**) the effect on lipid accumulation by staining with Oil Red is shown together with quantification of cell staining (right panel). Scale bar 125 μm. (**c**) HCT116 cells transfected with siRNA-Hakai2 or siRNA control in presence of absence of siRNA-FASN, analyzed by Western blot analysis and (**d**) staining with Oil Red together with quantification of cell staining results is. (**b** and **d**) Five pictures of each sample were taken and quantified. Values are means ± SD of staining intensity signal scoring per area. Quantification and calibration of the images were performed with Image Analysis Toolbox software for Image J. Kruskal–Wallis with Tukey correction test analyses show statistical differences between overexpression/silencing of Hakai or FASN and controls (*p < 0.05; **p < 0,01; ***p < 0,001; ****P < 0.0001). Scale bar 50 μm. (**e**) Immunohistochemistry staining (letf panel) and quantification (right panel) of FASN in mouse samples of the AOM/DSS Colitis-associated colorectal cancer model (inflamed epithelium and tumours, healthy mice, n = 7; AOM/DSS-treated mice, n = 14)**.** Immunohistochemistry staining and quantification were performed as indicated in figure legend 1. Scale bar 125 μm. The blots images were cropped prior hybridization and original blots are included in Supplementary Information.

Given the demonstrated role of Hakai controlling FASN protein at posttranslational level, we assessed whether Hakai may have an impact in lipid accumulation. For this purpose, HCT116 cells were first transfected with Flag-Hakai to analyze the effect of its overexpression in lipid accumulation. When Hakai is overexpressed (Fig. 5a), a decrease in lipid accumulation was confirmed by oil red staining (Fig. 5b). Moreover, lower FASN levels decreased lipid accumulation, while the dual knockdown of siRNA-FASN and siRNA-Hakai, did not allow to recover lipid accumulation supporting that Hakai may control lipid accumulation, at least in part, through FASN (Fig. 5c,d). Given the previous results showing that Hakai expression in AOM-DSS model of CAC was decreased in inflammatory conditions compared to tumour tissue of colitis-associated colorectal cancer (AOM DSS/Tumour) and healthy tissues, we extended our results to analyse FASN expression in this mouse model (Fig. 5e). An inverse expression of FASN expression with Hakai expression was detected in inflammatory AOM/DSS compared to tumour tissue of colitis-associated colorectal cancer and healthy tissues (Fig. 5e) further suggesting that Hakai may regulate FASN expression in mouse model of inflammatory bowel disease.

### FASN and Hakai expression in human IBD

**Figure 6:**
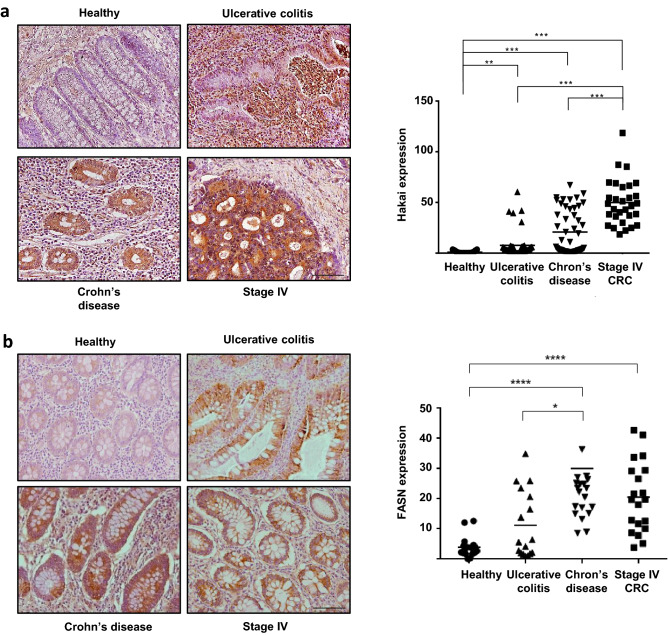
Expression of Hakai in human samples of UC, CD and colorectal adenocarcinoma stage IV and its healthy tissue pairs. (**a**) Immunohistochemistry staining of Hakai (left panel) and quantification (right panel) in healthy colonic mucosa, UC, CD and colorectal adenocarcinoma TNM-stage IV. Images obtained with 20 × objective. Scale bar 125 μm. (**b**) Staining intensity of FASN (left panel) and quantification (right panel) in healthy colonic mucosa, UC, CD and colorectal adenocarcinoma TNM-stage IV. (Normal colonic mucosa, n = 6, UC, n = 8; CD, n = 10; and colorectal adenocarcinoma, n = 6). Five pictures of each sample were taken and quantified. Data are represented as scatter plot. Values are means ± SEM of staining intensity signal scoring per area. Quantification and calibration of the images were performed with immunohistochemistry Image Analysis Toolbox software for Image J. Kruskal–Wallis with Tukey correction test analyses show statistical differences in colorectal cancer (TNM, SI-IV) respect to paired healthy samples (*p < 0.05; **p < 0,01; ***p < 0,001). Scale bar 125 μm.

Given that in the mouse model of inflammation-driven colorectal cancer, an inverse expression of Hakai and FASN expression was detected, we decided to analyse the expression of Hakai and FASN in human inflammatory colon disorders, including UC and CD. These two processes are chronic inflammatory conditions of the gastrointestinal tract that increase the risk of developing pre-neoplastic and neoplastic lesions. To compare the expression pattern of Hakai and FASN in the context of intestinal inflammation, human tissue samples from CAC, including UC and CD were analysed by immunohistochemistry. As shown in Fig. 6a, Hakai expression was significantly upregulated in UC and CD compared to normal tissues, and higher expression was detected compared to colorectal adenocarcinoma TNM-stage IV tissues. On the other hand, FASN expression was increased in CD samples compared to healthy tissues, but no significant differences were detected while comparing UC and healthy samples (Fig. 6b). Collectively, these results indicate that Hakai and FASN expression in the IBD mouse models do not mimic in the human IBD.

## Discussion

IBD is a chronic inflammatory disorder in gastrointestinal tract that increases the risk of developing colon cancer^39^. In the last years, important number of publications highlight the importance of protein ubiquitination in IBD^40,41^. The E3 ubiquitin-ligase Hakai, highly expressed in colon cancer tissues compared to adjacent normal tissues, plays a critical role during tumor progression^42^, however, the potential implication in IBD is still unknown. In the current study, we have shown that Hakai expression is downregulated in inflamed intestinal epithelium in different mouse models for IBD (Fig. 1), while higher expression was detected in tumor tissues from AOM-DSS CAC model (Fig. 1a). Furthermore, the Fatty Acid Synthase (FASN) is a novel Hakai-interacting protein identified in the present study. We show that Hakai induces FASN ubiquitination and degradation, thereby resulting in the downregulation of FASN-mediated fatty acid accumulation (Figs. 4, 5). The abnormalities in fatty acid metabolism have been reported in IBD, and are considered as one of the etiologies for the development of this disease^43^. By immunohistochemistry, we have shown that FASN expression increases in the mucosa in the mice with colitis. This observation agrees with our results and previous reported studies showing high FASN expression in patients with IBD and colorectal neoplasia^35,37^.

In our in vitro model system, we have demonstrated that Hakai overexpression induces FASN degradation, while Hakai-silencing increases FASN expression and, in consequence, fatty-acid accumulation, which is associated to the development of IBD. In the mouse model, Hakai expression is downregulated in inflamed intestinal epithelium accompanied to an increase expression of FASN. Although, several studies have reported the role of Hakai in different cancers^11,44,45^, to our knowledge, this is the first study that links Hakai with IBD. Other E3 ubiquitin-ligases were reported to be involved in IBD. For instance, RNF186 controls protein homeostasis in colonic epithelial and regulates intestinal inflammation. Indeed, RNF186 is expressed in colonic epithelial of the mice, and *Rnf186*^*−/−*^ mice are reported to lead to an increased risk of intestinal inflammation^46^. Accordingly, our results open the possibility to elucidate whether, in a similar manner, a decreased expression of Hakai in inflamed intestinal epithelia in mice may increase the risk of IBD. Importantly, in the tumors of the CAC mouse model, a higher expression of FASN and Hakai was detected, further supporting the implication of both proteins in colon cancer. However, in other studies, by gene expression prolife analysis, a decrease of FASN expression was described in patients with UC^47^. FASN was also described to play a crucial role in maintaining the homeostasis of intestinal barrier function, showing that loss of FASN in mouse intestine can initiate intestinal inflammation^48^. Intestinal homeostasis depends on multiple factor including the interaction between intestinal epithelium and the host immune system and the microbiota. Several regulatory mechanisms are reported to maintain intestinal homeostasis, and how the alteration of these pathways may precipitate the IBD^49^. Therefore, further studies are required in order to elucidate in which specific situation Hakai/FASN axis may contribute to intestinal homeostasis or IBD, as well as to reveal additional molecules that may help to orchestrate this regulatory process.

Previous studies have shown an increased ubiquitination mediated proteolysis in colonic biopsy samples from patients with IBD^8,9,50^. Indeed, other E3 ubiquitin ligases, such as RNF183, are reported to be up-regulated in the inflamed IBD mucosa, including UC and CD. RNF183 induces ubiquitination-mediated degradation of IκBα leading to the activation of NFκB-p65 in intestinal epithelial cells, further suggesting RNF183 role in NF-κB pathway in intestinal inflammation^51^. Hakai was recently reported to be associated to immune microenvironment regulation of periodontitis, a chronic inflammatory disease occurring in periodontal that involves complex interactions between pathogens and immune reactions^52,53^. This work demonstrates that Hakai is an important regulator of TNF and cytokine in immune reaction in periodontitis^53^. Given that TNF-α is a well-known inflammatory mediator that is highly expressed in the inflamed intestines of CD and UC patients^54^, it opens the possibility to explore whether Hakai may be linked to TNF-α signaling pathway in IBD.

In IBD human biopsies, Hakai expression was significantly upregulated in UC and CD compared to normal tissues, and higher expression was even detected in TNM-stage IV colorectal adenocarcinoma tissues. On the other hand, FASN expression was only increased in CD samples compared to healthy tissues, but no significant differences were detected between IBD and TNM-stage IV colorectal cancer. These results are not according to the ones observed in IBD mice, suggesting that Hakai regulation in mouse models does not accurately mimic human IBD, as observed in UC and CD. Although mouse models have been extensively used to study basic pathophysiological mechanisms, important controversies are reported regarding to how well murine models reflect human inflammatory diseases^55,56^. Indeed, Seok et at show that the genomic responses to different acute inflammatory stresses are highly similar in humans, but are not reproduced in the mouse models. Several factors can contribute to the differences seen in the molecular response of inflammatory mice and human disease including the evolutional and distance differences in cellular composition between mice and humans, as well as the complexity of the human disease^57–60^.

Taken together, we found that the E3 ubiquitin-ligase Hakai increases ubiquitination and degradation of FASN, thereby resulting in the regulation of FASN-mediated lipid accumulation, which is associated to the development of IBD. Further studies are needed to deepen into the role of Hakai in IBD in mouse models in order to better understand the regulatory mechanism and pathways that may influence intestinal homeostasis and its breakdown in IBD. Moreover, the role of Hakai/FASN axis in other diseases such as fatty liver disease awaits to be elucidated.

## Materials and methods

### Human intestinal tissues samples

Human biopsies from patients with UC and CD and colorectal cancer were obtained from the Pathological Anatomy Department from the “Complejo Hospitalario Universitario A Coruña” (CHUAC), under informed consent signed from all patients. Research investigation was approved by the Research Ethics Committee from A Coruña-Ferrol (Ethical Protocol Codes: 2018/257 and 2017/570) following standard ethical procedures of the Spanish regulation (Ley Orgánica de Investigación Biomédica, 14 July 2007) and according to the ethical standards in the 1964 Declaration of Helsinki. Paraffin samples were obtained from CHUAC Biobank integrated in the Spanish Hospital Platform Biobanks Network. Serial 4 µm sections from archived, formalin-fixed and paraffin-embedded intestinal tissue samples from patients (CD, n = 10; UC, n = 8 and colorectal cancer, n = 6) were analysed for Hakai protein expression.

### Mice intestinal tissues samples

Three different mouse models of CAC were used to analyze Hakai protein expression. First, a chronic inflammation-associated carcinogenesis mouse model from which biopsies were taken from C57BL/6J mice, in which the development of colorectal cancer associated with inflammation, was promoted through intraperitoneal administration a chemical carcinogen Azoxymethane (AOM) and the inflammatory agent, dextran sodium sulfate (DSS), was given with the drinking water as reported^29^. Second, a DSS model that mimics the acute colitis, on which only DSS is used in a single cycle of 8 days and concentration (3%). Animals were sacrificed 4 days after the end of the DSS cycle. Finally, an IL10^−/−^ model^61^, a genetically modified mice resulting in the enterocolitis appearance in presence of intestinal bacteria and imbalance in the function of the intestinal mucosa. All mouse intestinal tissue samples from C57BL/6J and C57BL/6 IL10 KO mice were kindly provided by Prof. Dr. Christoph Gasche (Medical University of Vienna, Austria).

### Microarray data mining in NCBI’s gene expression omnibus (GEO)

Data from Hakai expression obtained from a gene-centric and an experiment-centric perspective was obtained from the GEO Database (https://www.ncbi.nlm.nih.gov/sites/GDSbrowser/). All the data sets available were filtered looking for microarrays of models of colitis associated cancer. Exclusion criteria was common for the 3 searches and included mice models that could interfere in the evaluation of the physiological process, principally studies that evaluated the effect of any drug to prevent the development of the inflammatory process. For the AOM/DSS model subgroups were formed according to the exposure time to the AOM/DSS, which was 2 weeks for the inflamed tissue, 4 weeks for those with low dysplasia, between 6 and 8 weeks for high dysplasia and 20 weeks for CRC with the control group only treated with saline. For the other two models, acute colitis and IL-10 KO, criteria for subgroup formation were only case/control. The final studies selected (GDS4367, GSE31106 and GSE107810) were analysed using GEO2R (https://www.ncbi.nlm.nih.gov/geo/geo2r/), in order to identify genes differentially expressed across the experimental conditions. The results of the search for Hakai expression in the created subgroups are presented. Parameters for the GEO2R tool were not customized and were used as default.

### Antibodies and inhibitors

Hakai antibody (Invitrogen, Carlsbad, CA, USA) was used for western-blotting, and Hakai-2498 antibody used for immunohistochemistry was kindly provided by Dr. Fujita and Hakai antibody (Bethyl, Montgomery, TX, USA) was used for immunoprecipitation^12^. The rest of antibodies used for western-blotting are FASN antibody (Santa Cruz, Dallas, TX, USA), LC3 A/B antibody (Cell Signaling, Leiden, The Netherlands), E-cadherin antibody (BD Trans Lab, Franklin Lakes, NJ, USA), β-catenin antibody (Cell Signaling, Leiden, The Netherlands), GAPDH antibody (Invitrogene) and mouse and rabbit secondary antibodies (GE Healthcare, Chicago, IL, USA). Proteasome inhibitor MG132 (Sigma-Aldrich, St. Louis, MO, USA) was added for 6 h using 10 µM and 30 µM. Lysosome degradation inhibitor Chloroquine (Sigma-Aldrich, St. Louis, MO, USA), was added for 24 h at 50 µM and for 6 h at 100 µM. Autophagy inhibitor 3-Methyladenine (Sigma-Aldrich, St. Louis, MO, USA) was added for 24 h at 5 mM and 10 mM. Protein synthesis inhibitor cycloheximide (Sigma-Aldrich) was used at 10 μg/mL for the indicated times.

### Immunohistochemistry

For immunohistochemistry, slides containing sections of mice and human intestinal tissues (4 µm) were deparaffinised, rehydrated and processed as previously described^31^. Incubation with primary antibody was carried out in a wet chamber overnight at 4 °C. Hakai dilution was 1:700 and FASN dilution was 1:500. Commercial kit for immunohistochemistry was Dako EnVision + System, Peroxidase (EnVision + System, HRP) purchased to Agilent Technologies, Inc. Nuclei were counterstained with Gill´s haematoxylin and mounted with DePeX. Pictures were taken with an Olympus BX50 microscope. Quantification of HRP signals was performed with ImageJ software by analysing 5 photographs of each sample, and the represented results are shown as mean ± SEM.

### Cell culture, transfection and construction of lentivirus and inducible cell lines

HCT 116 cell line was cultured in Dulbecco’s Modified Eagle Medium (DMEM) and HT29 cell line was cultured in McCoy's 5A medium. All media were supplemented with 1% penicillin/streptomycin and 10% of heat-inactivated fetal bovine serum (FBS). All cell lines were grown at 37 °C in a humidified incubator with 5% of CO_2_. Cells were regularly tested for mycoplasma. Plasmids used for transfection were pcDNA-Flag-Hakai, pBSSR-HA-Ubiquitin and pSG-v-Src which were kindly provided by Dr. Fujita (University College London, UK). Transient transfection for Hakai silencing was performed by employing two different siRNA oligonucleotides for Hakai: Hakai-1 (5′-CTCGATCGGTCAGTCAGGAAA-3′) and Hakai-2 (5′-CACCGCGAACTCAAAGAACTA-3′) or siRNA FASN oligonucleotide (5′-GCUACAUGGCCCAAGGGAA-3′). Transfection experiments were performed by using Lipofectamine 2000 Transfection Reagent and Opti-MEM (Thermo Fisher Scientific) media following manufacturer’s protocol. Universal Non-coding siRNA (Sigma-Aldrich, St. Louis, MO, USA) was used as a negative control of transfection. Lentiviral vector system for Hakai expression (SMARTvector Inducible Lentiviral shCBLL1) by doxycycline inducible sh-RNAs particles were acquired in Dharmacon (Horizon Perkin Elmer Group). Lentiviruses were propagated using previously described methods^62^. Doxycycline (Sigma) was added to the medium at final concentration of 1 µg/ml, and cells were incubated for 72 h. Two different clones of HT29 colon cancer cells transduced with viral supernatant, containing sh-Hakai (sh-Hak1 and sh-Hak2) or sh-control (sh-), were selected and Hakai knockdown efficiency was monitored by western blot.

### Interactome analyses

HCT116 cells were used for interactome analysis. Cell pellets were incubated with lysis buffer (20 mM Tris–HCl pH 7,5, 150 mM NaCl and 1% Triton X-100, 125 mg/mL N-ethylmaleimide) in rotation at 4 °C for 30 min. Pre-clearing was performed to eliminate unspecific interactions. For large scale immunoprecipitation, cells were used per point of the experiment (IgG and IP). 40 μL of Dynabeads protein A (Thermo Fisher Scientific) were resuspended in filtered PBS-T 0,1% and incubated with 5 μg of Hakai Bethyl antibody or control IgG 2 h at 4 °C on rotation for beads-antibody coupling. Then, beads and protein sample were incubated overnight at 4 °C on rotation. Samples were loaded into SDS-PAGE gel and band was stained with Sypro-Ruby (Lonza) fluorescent and processed to in gel digestion as previously described^63^. Samples were reduced in 10 mM dithiothreitol and dissolved in 50 mM ammonium bicarbonate (AMBIC) (Sigma-Aldrich). Then samples were alkylated with iodoacetamide 55 mM dissolved in AMBIC 50 mM (Sigma-Aldrich). The gel pieces were rinsed with AMBIC 50 mM in 50% methanol (HPLC grade, Scharlau) and acetonitrile (HPLC grade, Scharlau) was added for dehydration. Finally, they were dried in a SpeedVac (Thermo Fisher Scientific) and digested with porcine trypsin (Promega) to a final concentration of 20 ng/µL in AMBIC 20 mM for a final overnight incubation at 37 °C. For mass spectrometric analysis, samples were processed by the Proteomics Platform of Biomedical Research Institute of Santiago de Compostela (IDIS). The separation of peptides was done by Reverse phase chromatography. The 400 micro nanoLC liquid chromatography system (Eksigent Technologies, ABSciex) combined with a Triple Time-of-flight (TOF) 6600 high speed mass spectrometer (ABSciex) were used for creating a gradient. Analysis of peptides was performed on the C18CL reverse phase column (150 × 0.30 mm, 3 µm, 120 Å) (Eksigent, ABSciex). The peptides were separated using a 90-min gradient ranging from 2 to 90% of mobile phase B (mobile phase A: 0.1% formic acid, 2% acetonitrile; mobile phase B: 0.1% formic acid, 100% acetonitrile). Acquisition of data was performed on a Triple TOF 6600 system (ABSciex). The TF 1.7.1 analyst software was used for instrument operation (ABSciex). A switching criterion was used for ions greater than the mass/charge ratio (m/z) 350 and less than m/z 1400, with a mass tolerance of 250 ppm, a charge state of 2–5 and a threshold of abundance of more than 200 accounts (cps). The computer analysis of the raw data obtained was carried out using ProteinPilotTM 5.0.1 software (ABSciex). Analysis was performed with the Significance Analysis of INTeractome (SAINT) score SAINTexpress^64^. Preys with SAINT probability score cut-off of 1 detected by at least two exclusive spectral counts were deemed high confidence Hakai-interacting proteins.

### Western blot analysis, real-time quantitative PCR (RT-qPCR) and immunofluorescence staining

For western blot analysis, whole cell extracts were obtained as described previously^31^. For RT-qPCR, total RNA was extracted using TriPure isolation reagent (Roche,Basel, Switzerland). mRNA levels were analysed in technical triplicates by quantitative RT-PCR, following specifications of reverse retrotranscriptase kit (NZYTech, Lisbon, Portugal). Amplification was performed in a Light Cycler 480 (Roche, Basel, Switzerland) and data was analysed by qBase + analysis software (Biogazelle, Zwijnaarde, Belgium). Primers used for FASN were F′ TTCTACGGCTCCACGCTCTTCC and R′ GAAGAGTCTTCGTCAGCCAGGA. As housekeeping control HPRT primers F′-TGACCTTGATTTATTTTGCATACC and R′-CGAGCAAGACGTTCAGTCCT or RPL13A F′-CTCAAGGTGTTTGACGGCATCC and R′-TACTTCCAGCCAACCTCGTGAG were used. Immunofluorescence was performed as described^31^. Briefly, cells were fixed with PFA 4% for 15 min and permeabilized with 0.5% Triton X-100/PBS for 15 min. Primary antibodies were incubated for 2 h, at RT and secondary antibody was incubated at RT for 1 h. Nuclear staining was performed using 1:10,000 Hoechst dilution (Life Technologies, Carlsbad, CA, USA). ProLong Gold Antifade Mountant (Life Technologies, Carlsbad, CA, USA) was employed for coverslips mounting. Images were obtained by using fluorescence microscope “Monitorized reflected Fluorescence System” (Olympus).

### Immunoprecipitation and ubiquitination assays

For immunoprecipitation experiments, cells were lysed for 20 min in 1 ml of 1% Triton X-100 lysis buffer (20 mM Tris–HCl pH 7.5, 150 mM NaCl and 1% Triton X-100) containing 10 μg/ml leupeptin, 10 μg/ml aprotinin and 1 mM phenylmethanesulphonyl fluoride (PMSF). Supernatants were immunoprecipitated for 2 h with 2 μg of anti-Hakai antibody or anti-HA bound to protein G PLUS-Agarose beads (Santa Cruz Biotechnology, USA), followed by SDS–polyacrylamide gel electrophoresis (PAGE) and western blotting with the indicated antibodies as previously reported. For ubiquitination assays, HCT116 cells were transfected with 0.25 µg Src, 0.75 µg Flag-Hakai, and 0.5 µg HA-ubiquitin using Lipofectamin 2000 (Invitrogen, UK). Cell extracts were obtained in the above lysis buffer used for immunoprecipitation, supplemented with 10 mM N-ethylmaleimide.

### Oil-red staining assay

HCT116 and HT29 cells (10^5^) were seeded in 8-well chambers (Millicell EZ SLIDE 8-well glass, Millipore) were washed with 1 × PBS and stained with a freshly prepared Oil Red O (ORO) working solution as described previously^33^. Staining was observed using light microscope (Olympus BX61). Positively-stained areas were used to quantify lipid accumulation using ImageJ software by analysing 5 photographs. The represented results are shown as mean ± SEM.

### Statistical analysis

Shapiro–Wilk test was used to check a normal distribution and Levene test to assess the equality of variances. Statistical significance of data was determined by applying a two-tailed Student t-test or ANOVA depending on the data. Results obtained are expressed as mean ± SD or mean ± SEM. Quantification of human IHQ did not follow a normal distribution therefore we used Kruskal–Wallis with Tukey correction test. Significant differences among the experimental groups indicated in the figures is shown as *P < 0.05, **P < 0.01 ***P < 0.001 and ****P < 0.0001.

## Supplementary Information

Supplementary Figures.

## Data Availability

The datasets generated and/or analysed during the current study are available in the ProteomeXchange repository, accession number PXD029136. Full blots are included in Supplemental Figure file.

## Abbreviations

CAC: Colitis-associated cancer
CD: Crohn’s disease
IBD: Inflammatory bowel diseases
UC: Ulcerative colitis
