# Supplementary material for: Role of the E3 ubiquitin-ligase Hakai in intestinal inflammation and cancer bowel disease
Source: Sci Rep. 2022 Oct 20;12:17571. doi: 10.1038/s41598-022-22295-w (PMC9584894; doi:10.1038/s41598-022-22295-w)
Supplement: Supplementary file 1 — Supplementary Figures. [file 41598_2022_22295_MOESM1_ESM.pdf]

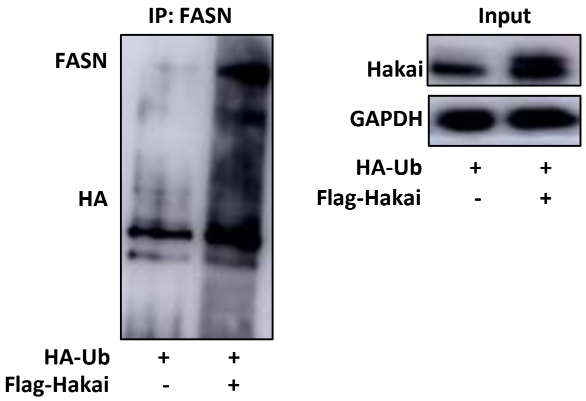

Fig. S1. Hakai-dependent ubiquitination of FASN Flag-Hakai and HA-ubiquitin were transiently transfected into HCT116 cells. Immunoprecipitation was performed with the anti-FASN antibody before Western blotting, using the indicated antibodies. Please note that the blot at the left panel was firstly blotted using HA-antibody to detect ubiquitin and then re-blotted using FASN antibody.

Fig. S1.

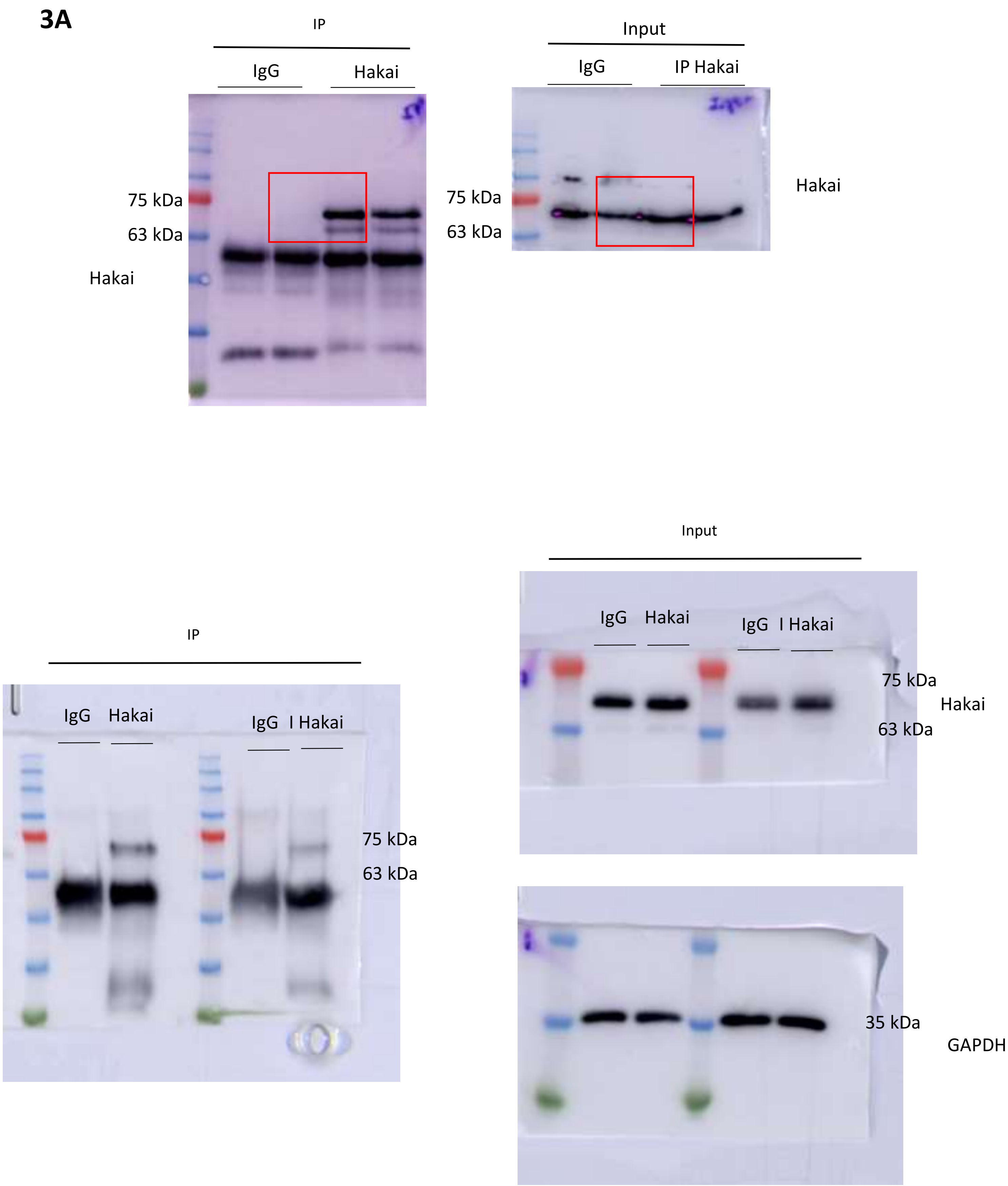

Fig. S2. Full blots corresponding to Figure 3A

3C

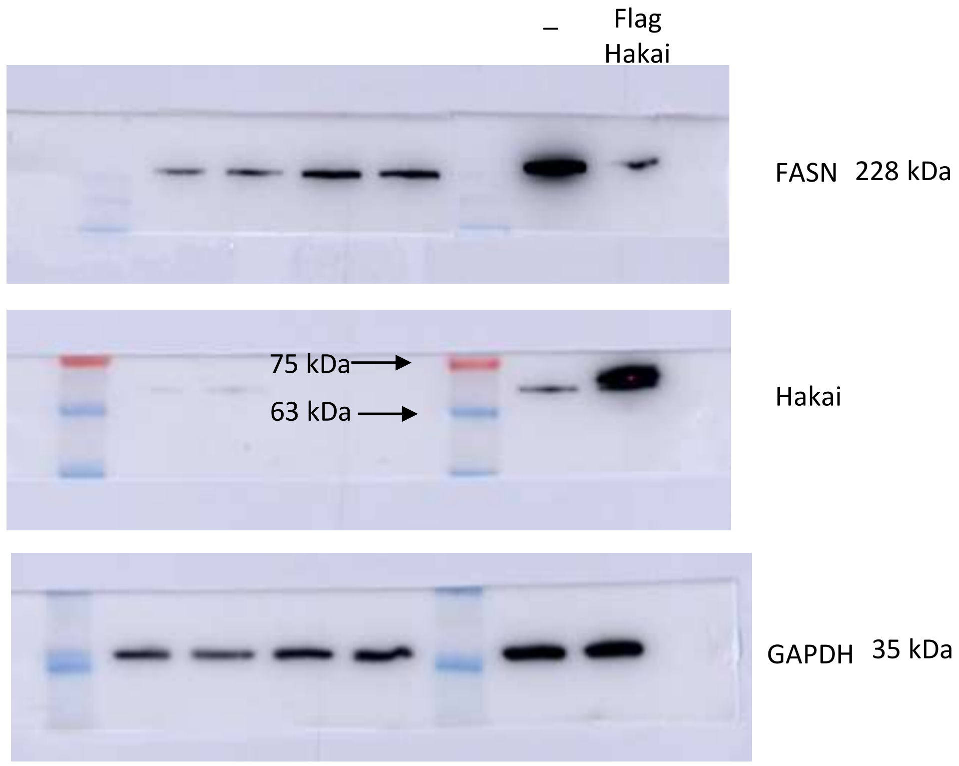

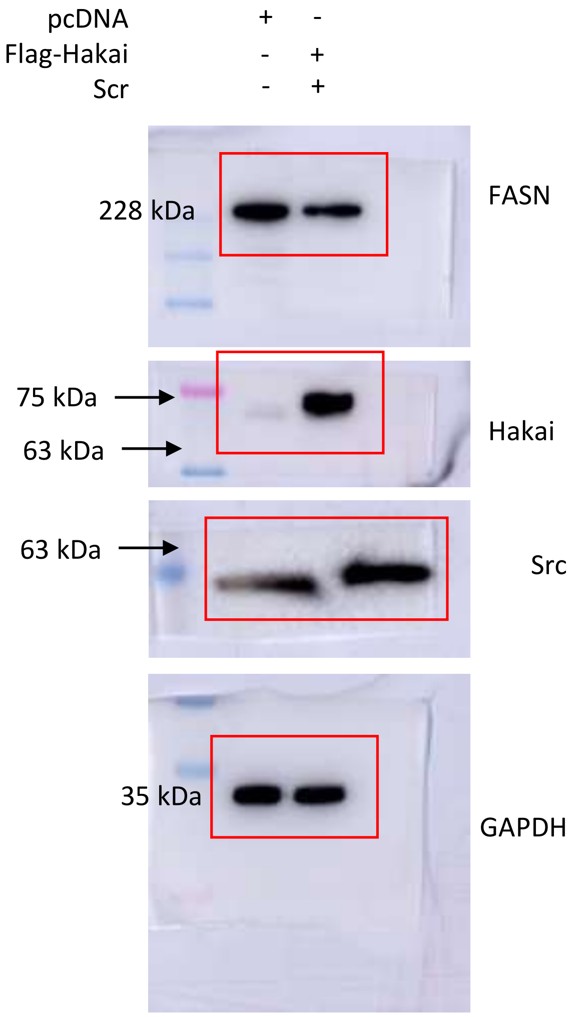

Fig. S3. Full blots corresponding to Figure 3C

3E

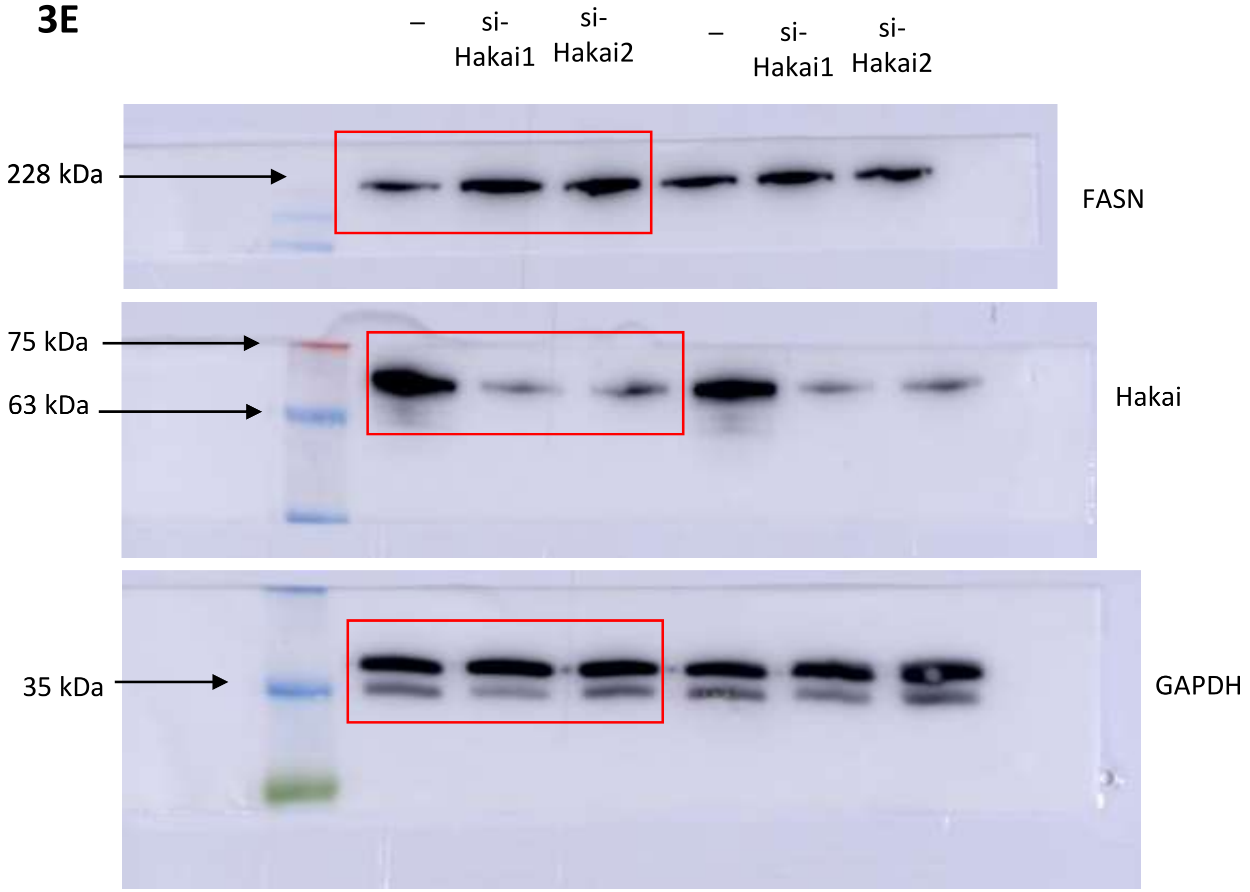

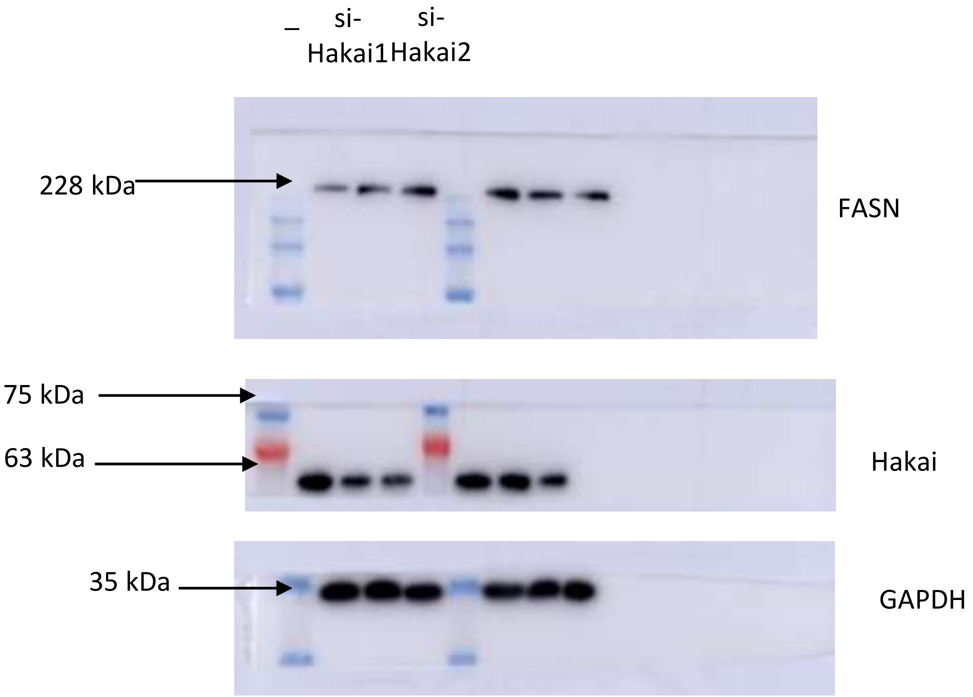

Fig. S4. Full blots corresponding to Figure 3E

3F

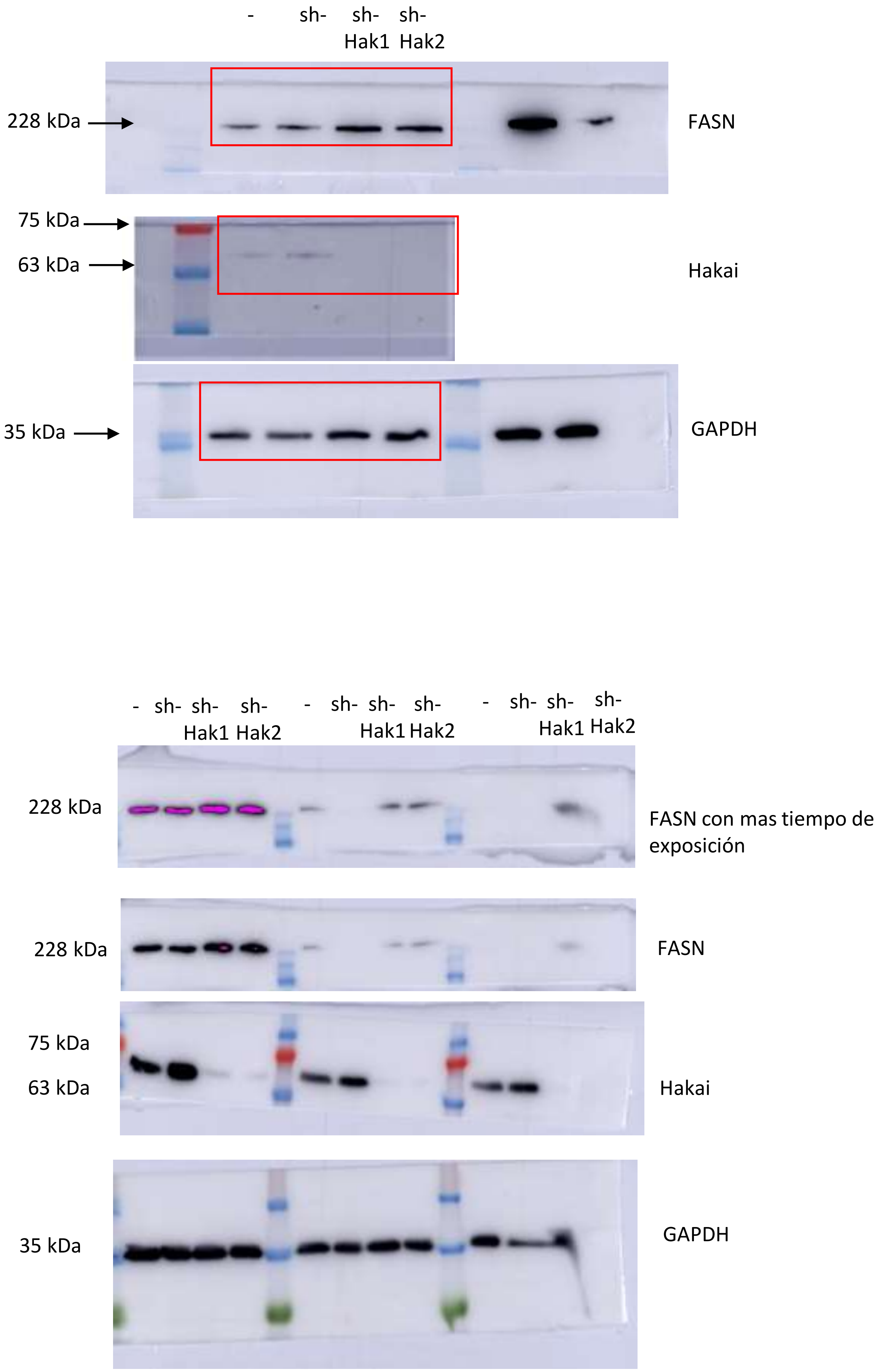

Fig. S5. Full blots corresponding to Figure 3F

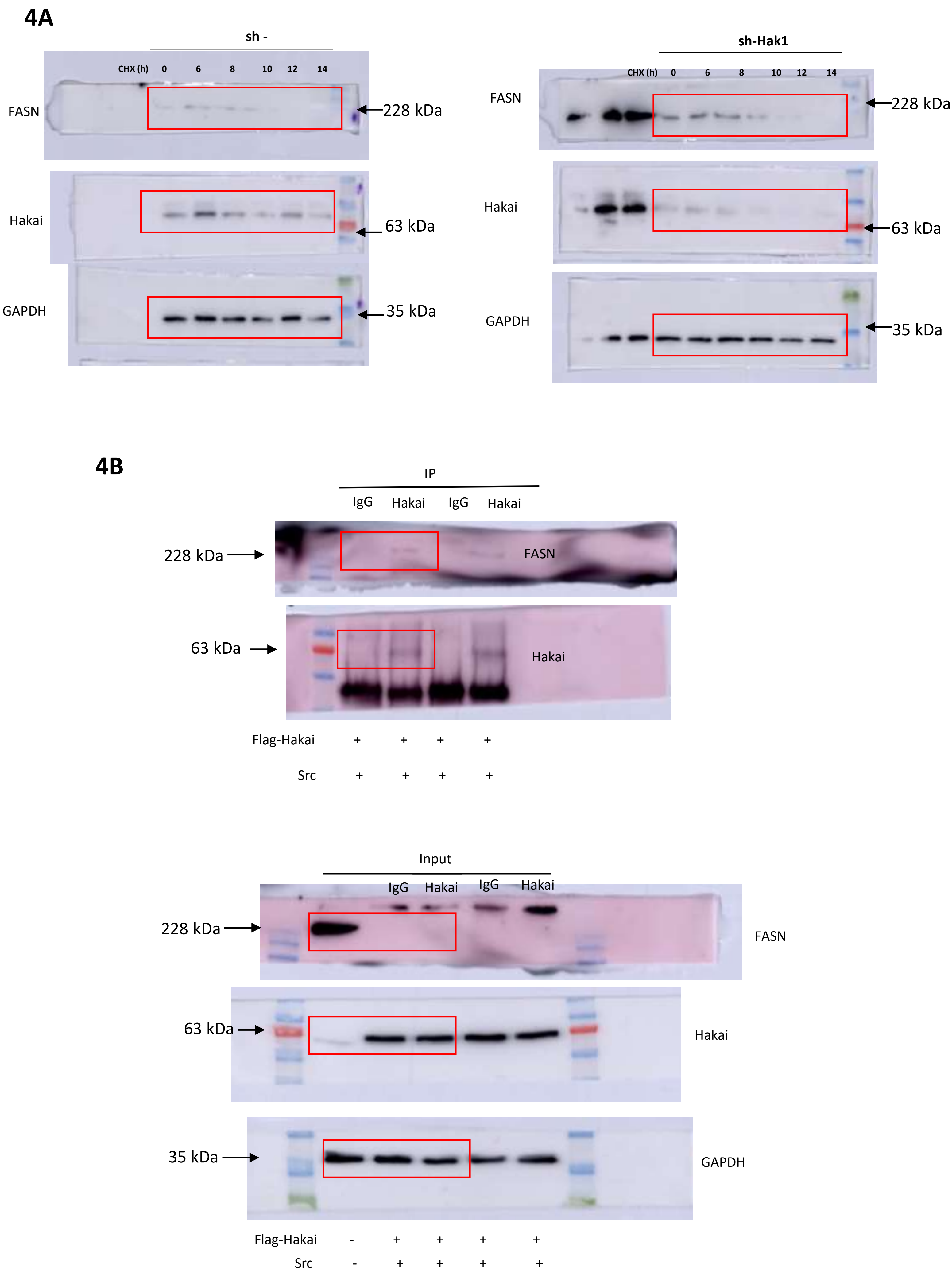

Fig. S6. Full blots corresponding to Figure 4A and B

4C

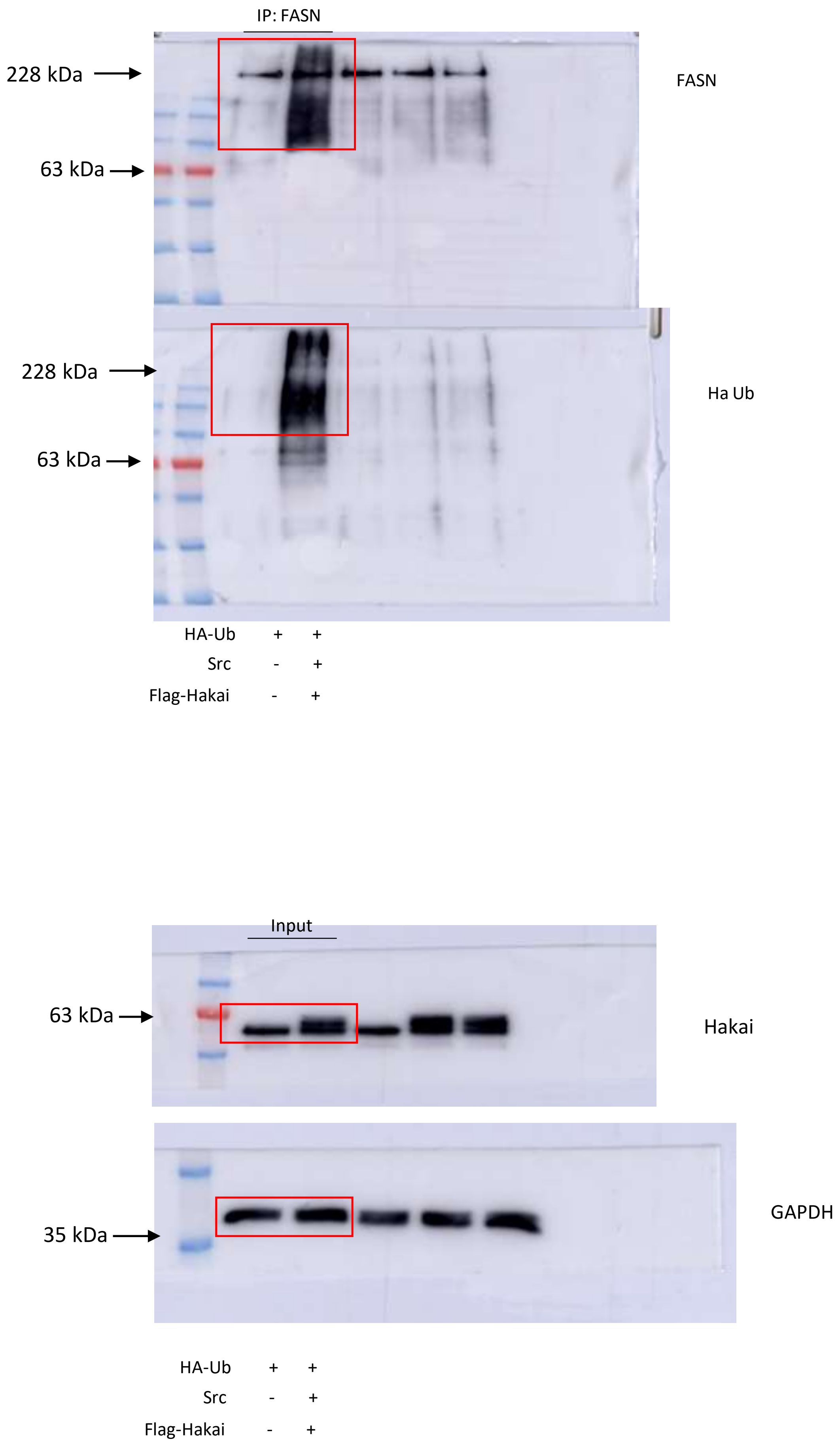

Fig. S7. Full blots corresponding to Figure 4C

4C

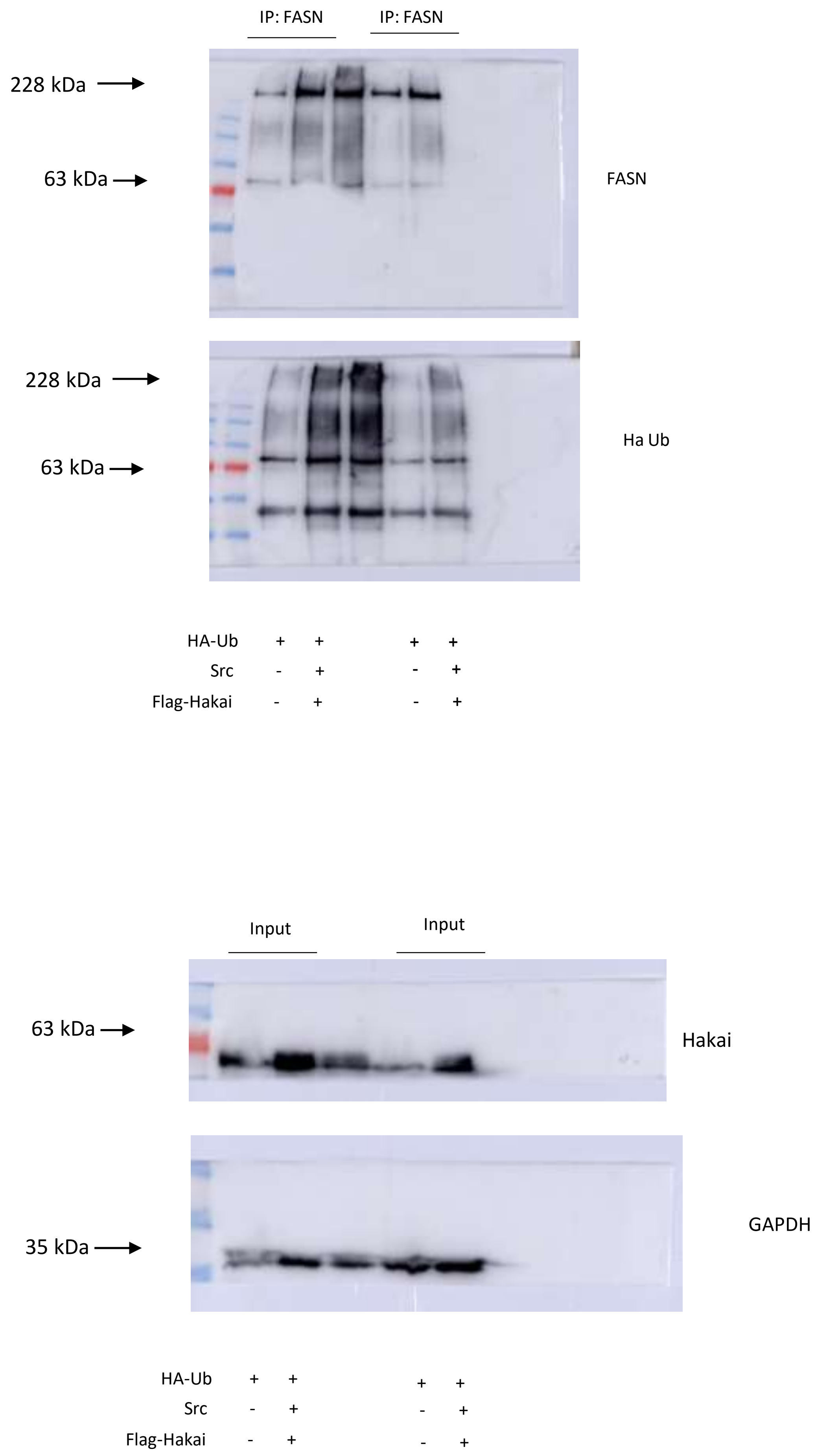

Fig. S8. Full blots corresponding to Figure 4C

4D

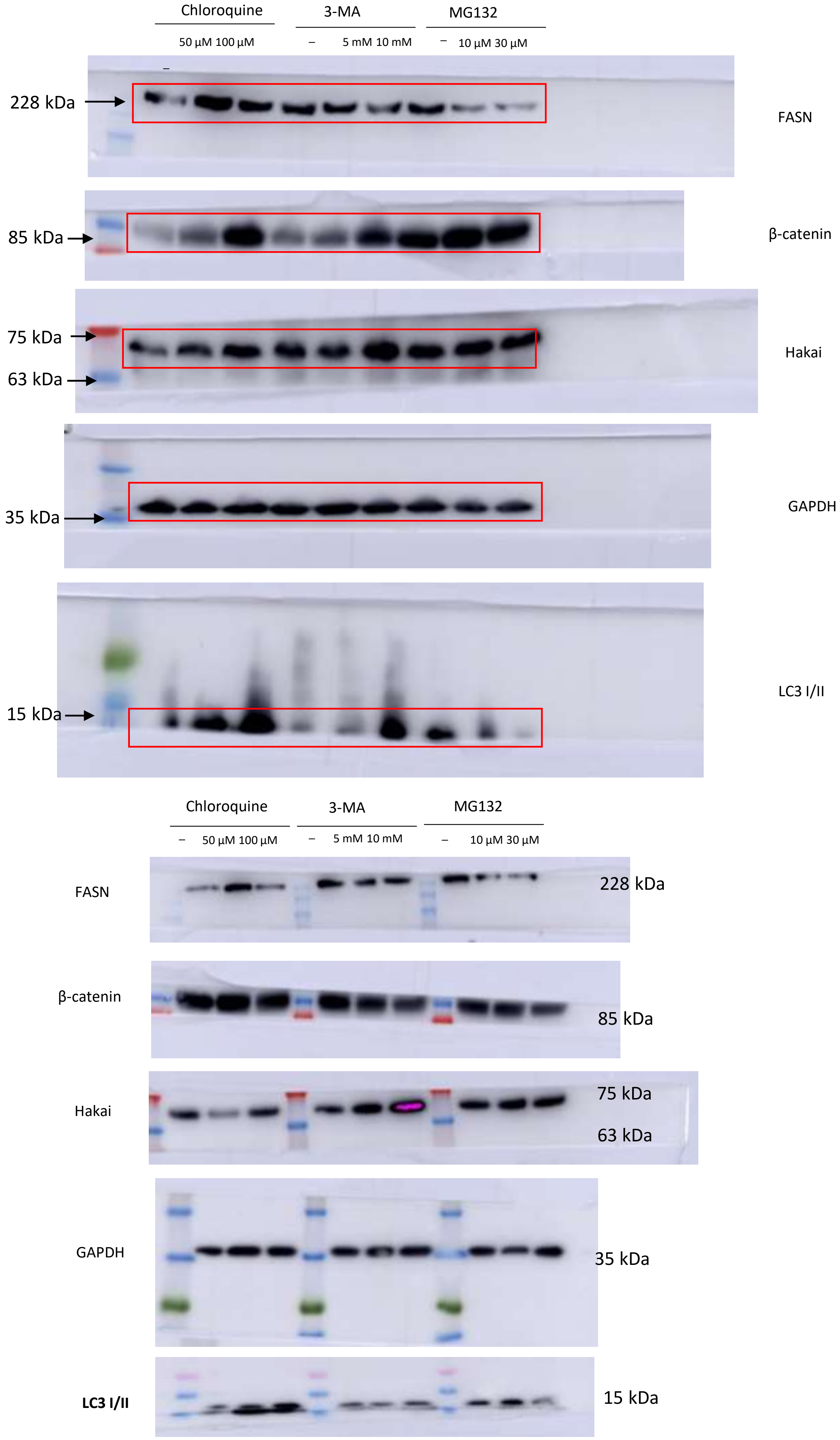

Fig. S9. Full blots corresponding to Figure 4D

4D

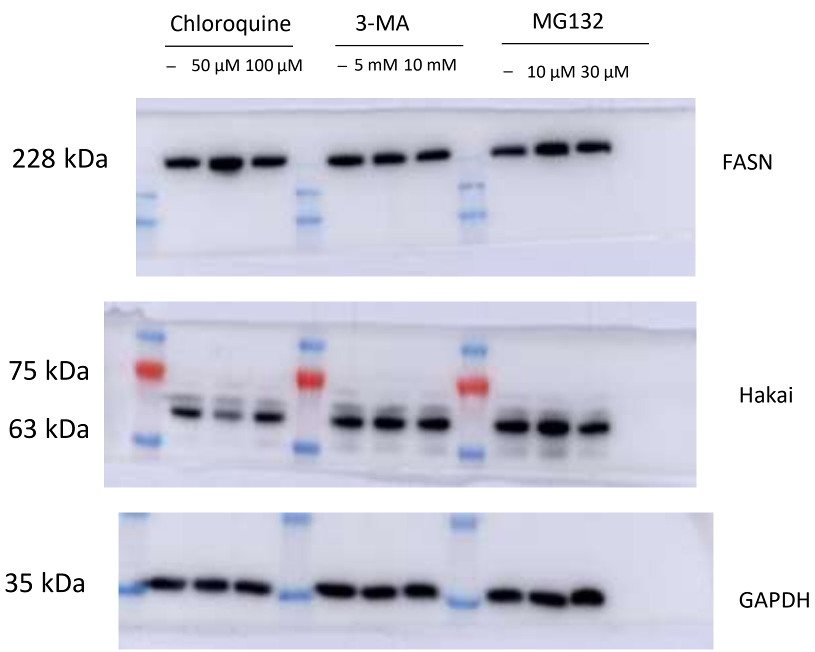

4E

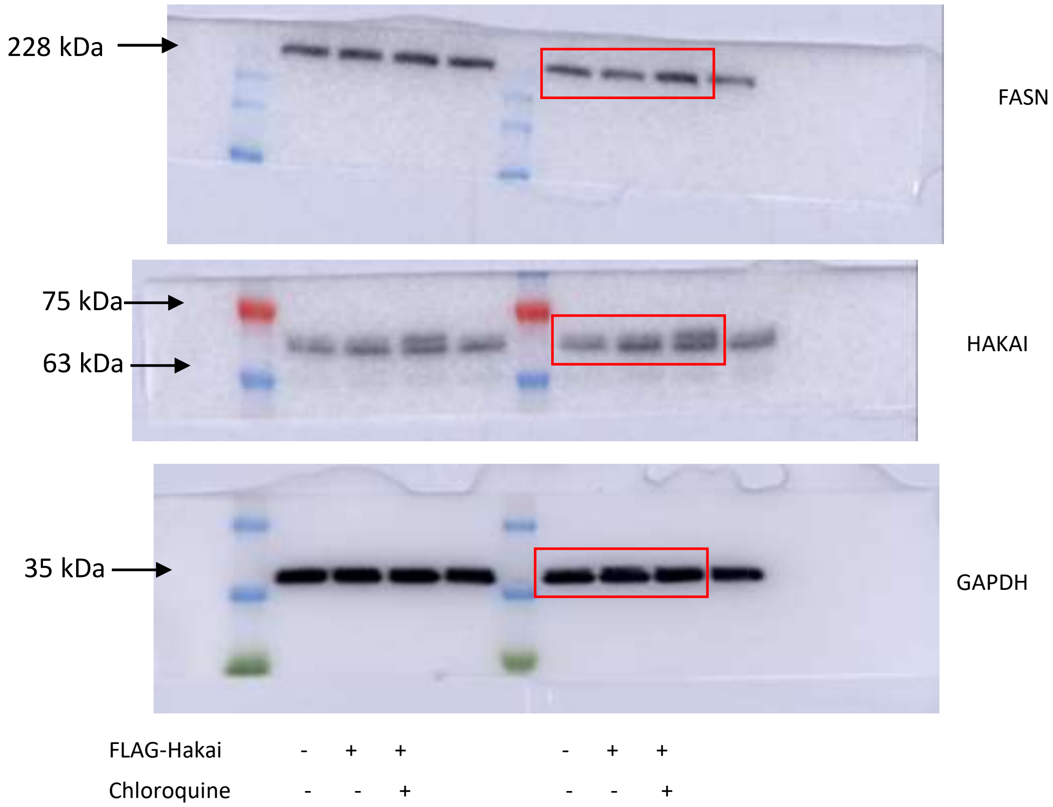

Fig. S10. Full blots corresponding to Figure 4D and E

4E

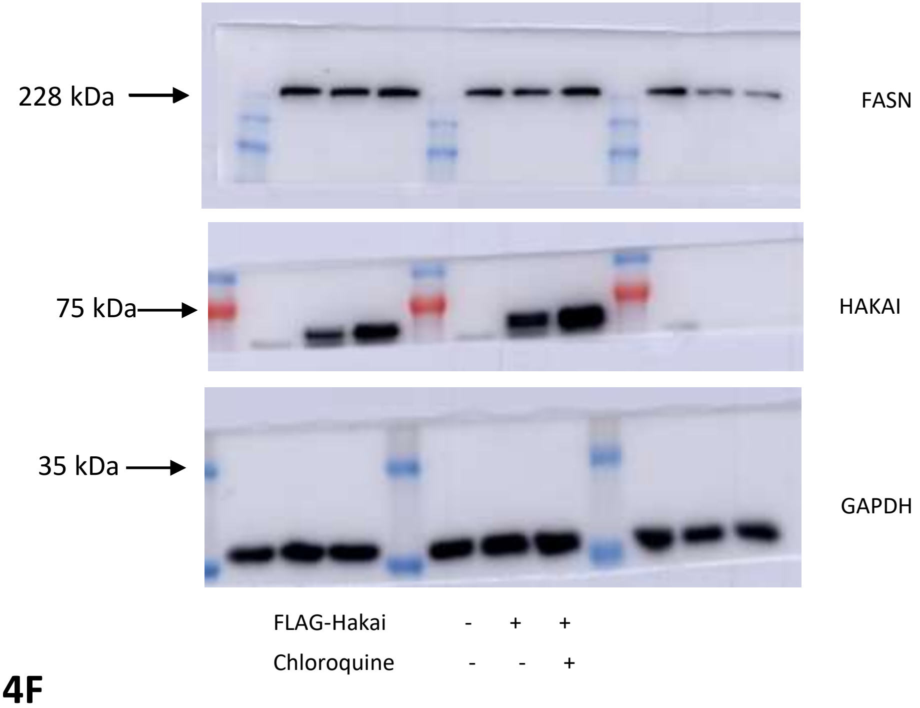

4F

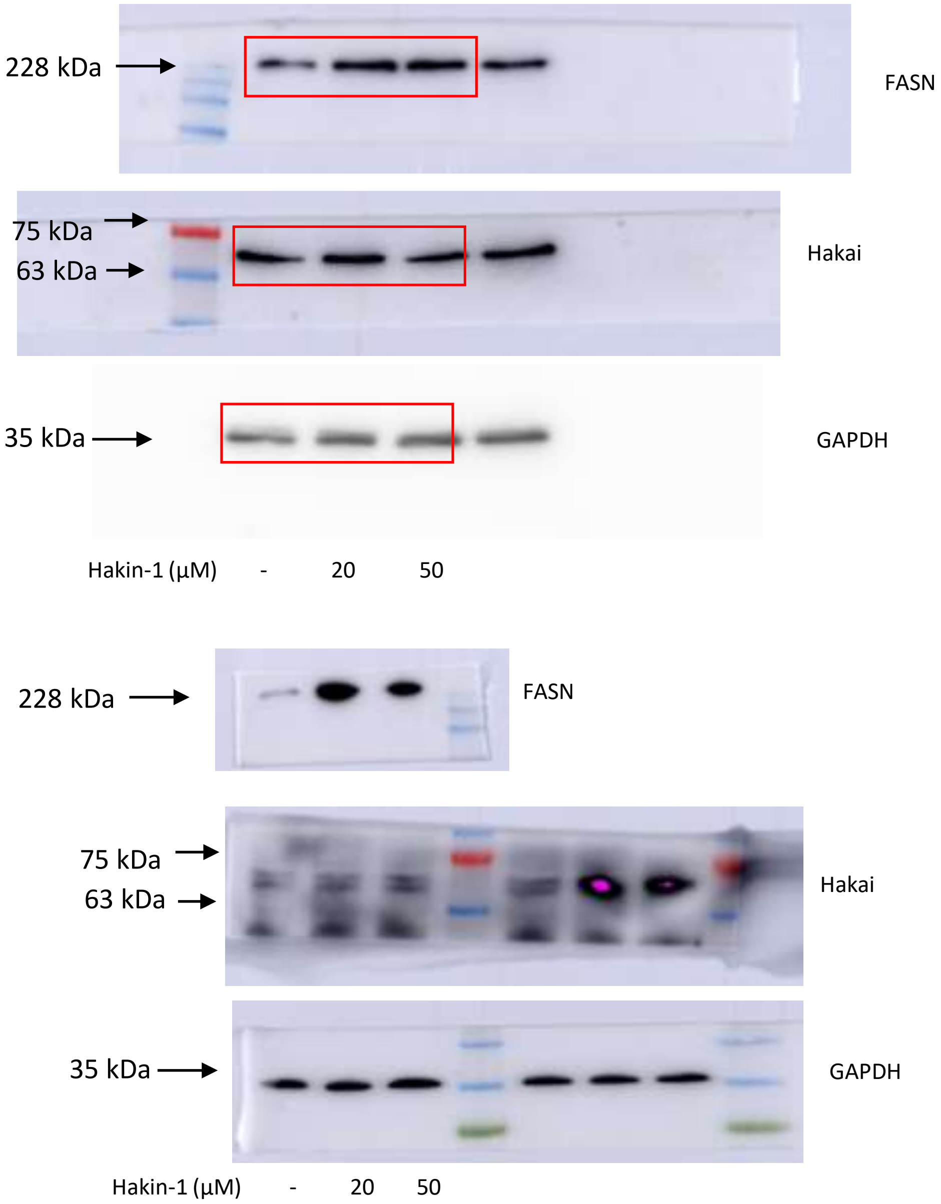

Fig. S11. Full blots corresponding to Figure 4E and F

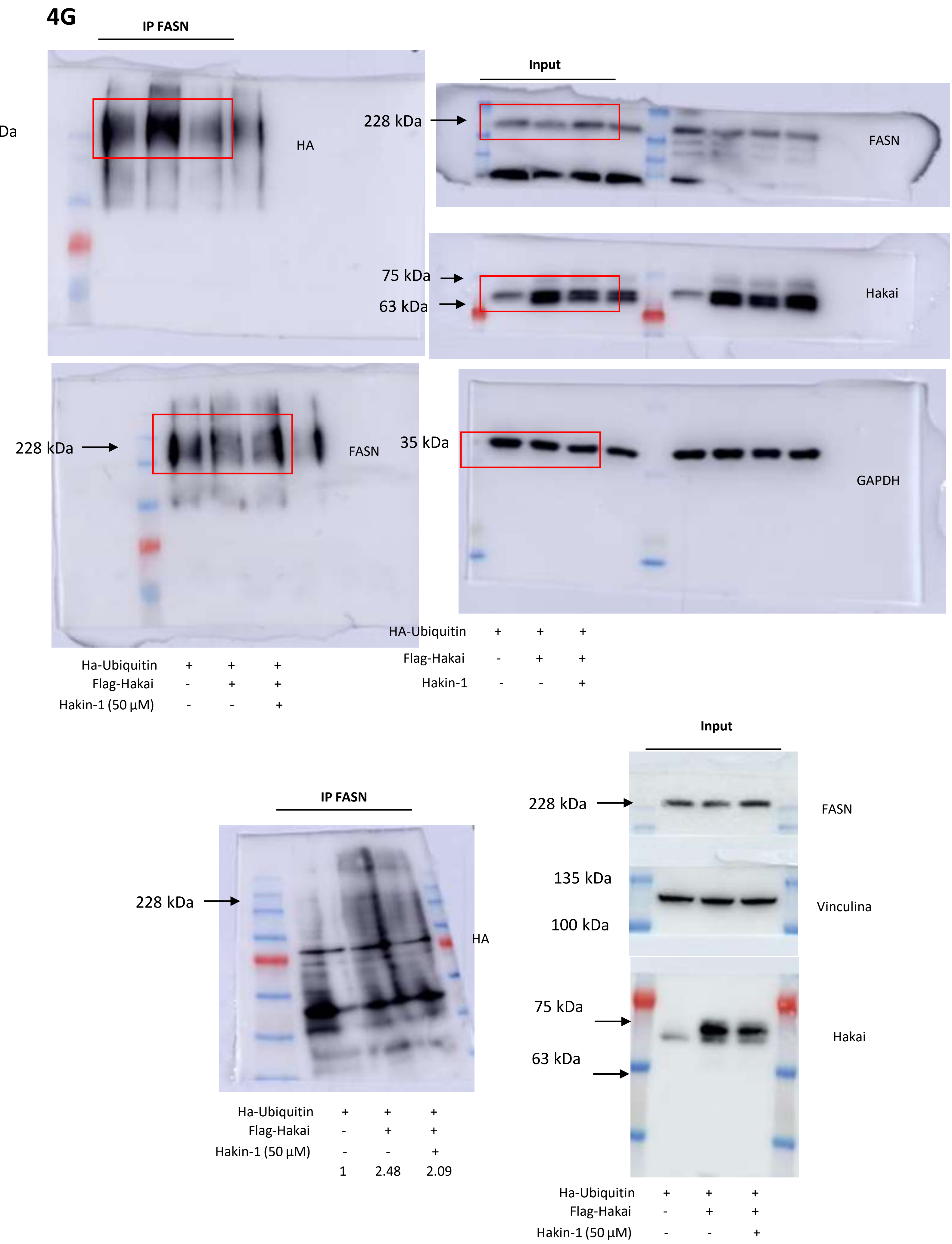

Fig. S12. Full blots corresponding to Figure 4G

5A

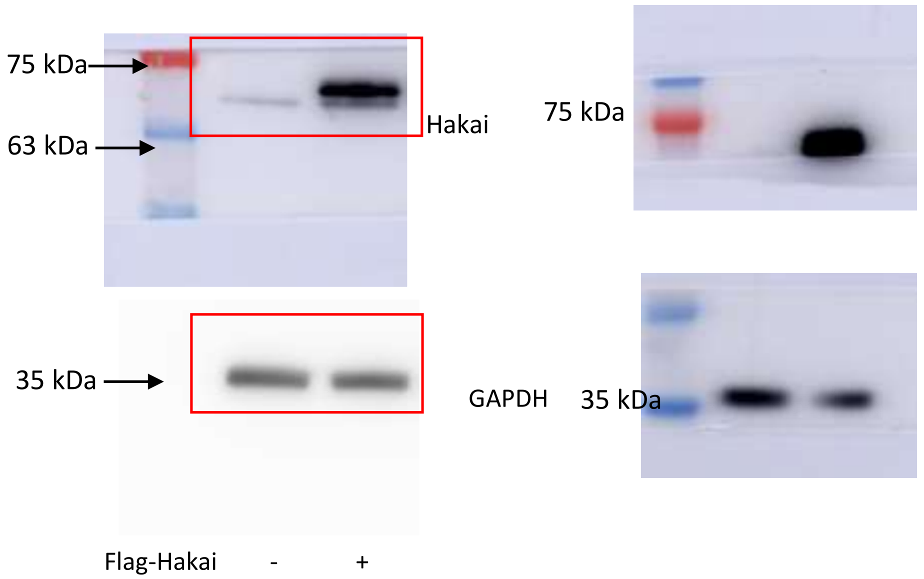

5C

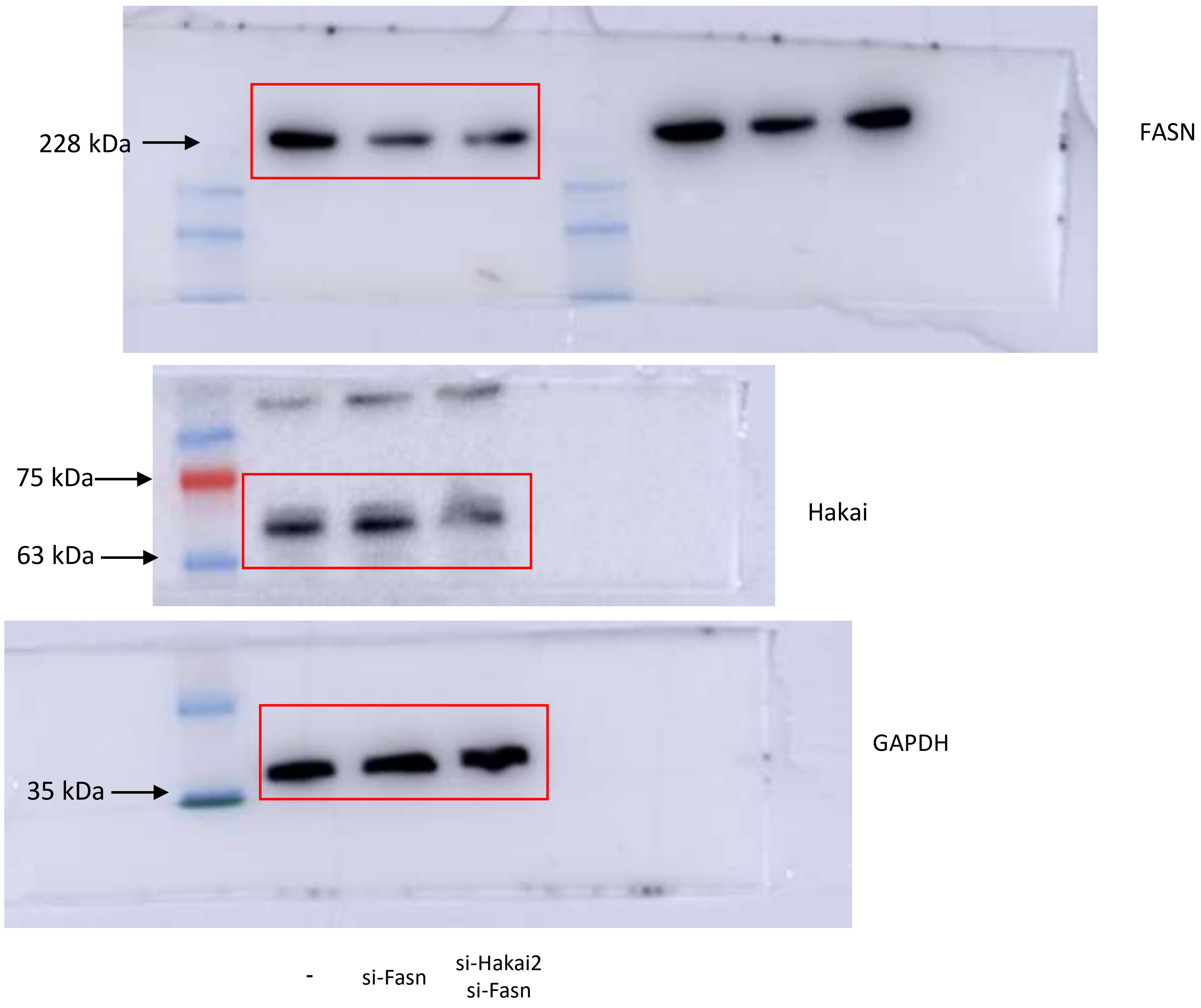

Fig. S13. Full blots corresponding to Figure 5A and C

5C

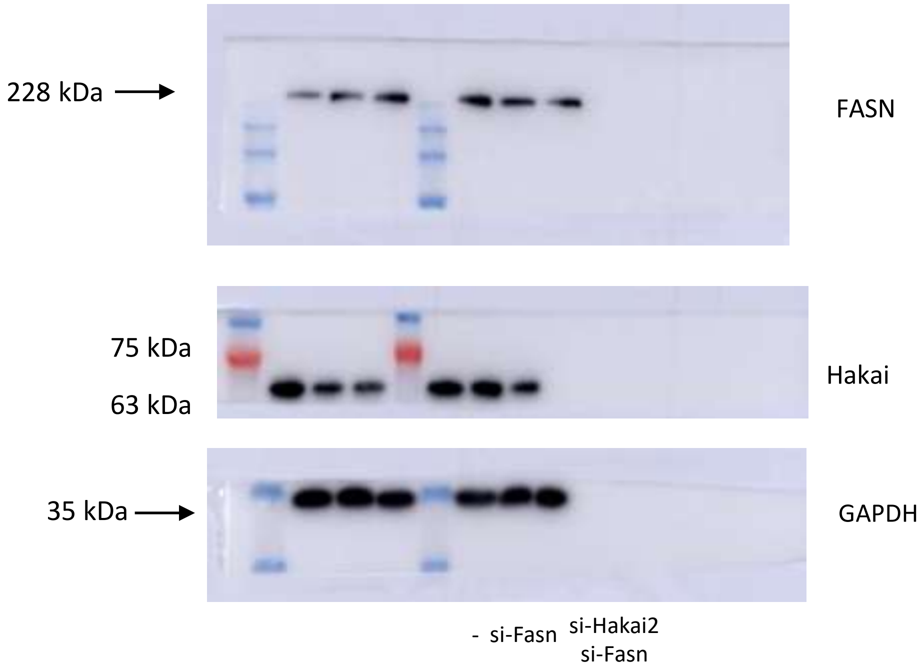

S1

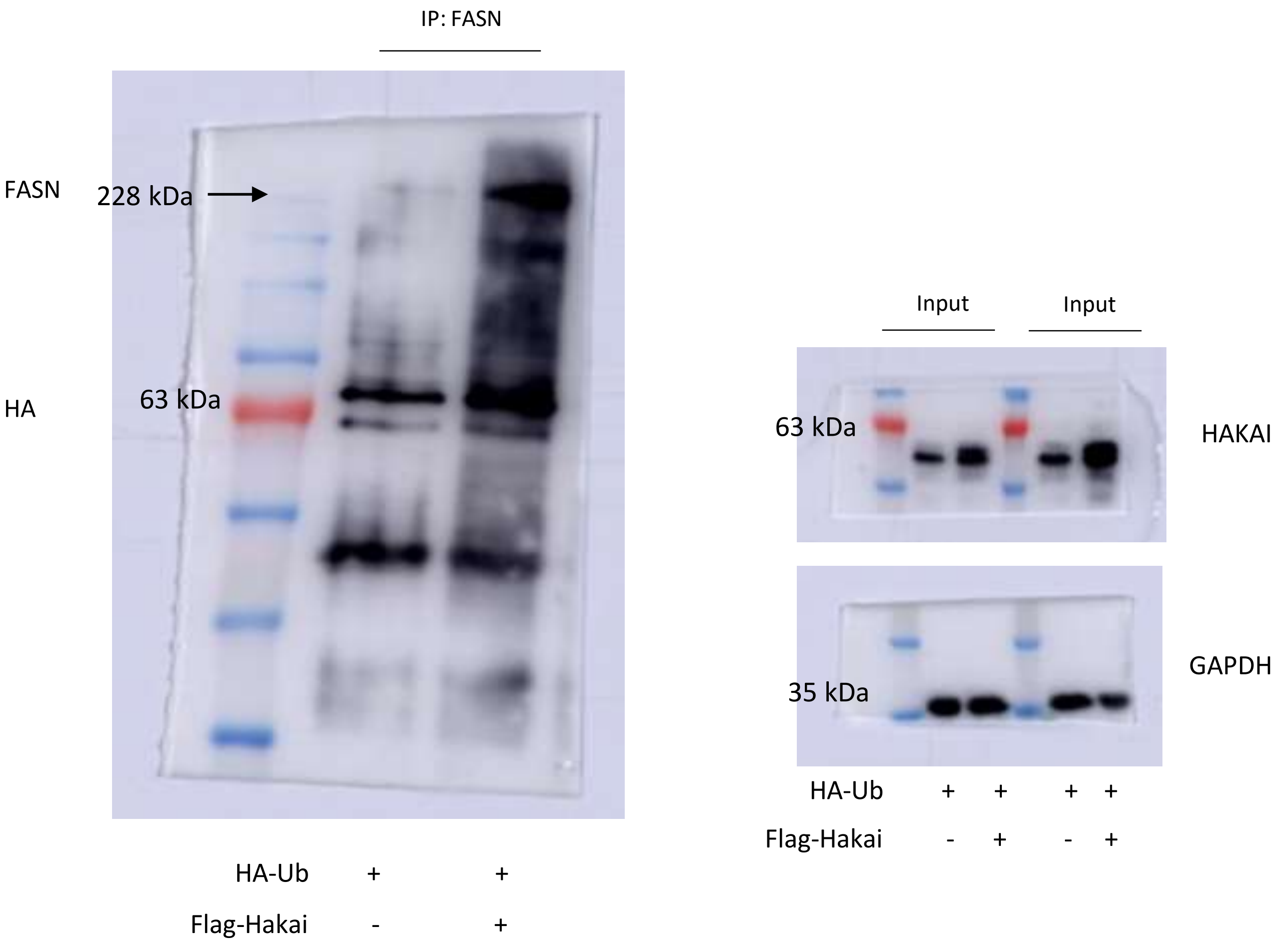

Fig. S14. Full blots corresponding to Figure 5 C and Fig S1
